# Dissolution and solubility of calcite-rhodochrosite solid solutions [(Ca_1-x_Mn_x_)CO_3_] at 25 °C

**DOI:** 10.1186/s12932-021-00075-1

**Published:** 2021-05-26

**Authors:** Yinian Zhu, Peijie Nong, Nan Mo, Zongqiang Zhu, Huan Deng, Shen Tang, Hongqu Yang, Lihao Zhang, Xingxing Wang

**Affiliations:** 1 College of Environmental Science and Engineering, Guilin University of Technology, Guilin, 541004 China; 2 Guangxi Key Laboratory of Environmental Pollution Control Theory and Technology, Guilin University of Technology, Guilin, 541004 China; 3 Collaborative Innovation Center for Water Pollution Control and Water Safety in Karst Area, Guilin University of Technology, Guilin, 541004 China

**Keywords:** Calcite, Rhodochrosite, Solid solution, Dissolution, Solubility, Lippmann diagram

## Abstract

A complete series of calcite-rhodochrosite solid solutions [(Ca_1-x_Mn_x_)CO_3_] are prepared, and their dissolution processes in various water samples are experimentally investigated. The crystal morphologies of the solid solutions vary from blocky spherical crystal aggregates to smaller spheres with an increasing incorporation of Mn in the solids. Regarding dissolution in N_2_-degassed water, air-saturated water and CO_2_-saturated water at 25 °C, the aqueous Ca and Mn concentrations reach their highest values after 1240–2400 h, 6–12 h and < 1 h, respectively, and then decrease gradually to a steady state; additionally, the ion activity products (log_IAP) at the final steady state (≈ solubility products in log_*K*_sp_) are estimated to be − 8.46 ± 0.06, − 8.44 ± 0.10 and − 8.59 ± 0.10 for calcite [CaCO_3_], respectively, and − 10.25 ± 0.08, − 10.26 ± 0.10 and − 10.28 ± 0.03, for rhodochrosite [MnCO_3_], respectively. As X_Mn_ increases, the log_IAP values decrease from − 8.44 ~ − 8.59 for calcite to − 10.25 ~ − 10.28 for rhodochrosite. The aqueous Mn concentrations increase with an increasing Mn/(Ca + Mn) molar ratio (X_Mn_) of the (Ca_1-x_Mn_x_)CO_3_ solid solutions, while the aqueous Ca concentrations show the highest values at X_Mn_ = 0.53–0.63. In the constructed Lippmann diagram of subregular (Ca_1-x_Mn_x_)CO_3_ solid solutions, the solids dissolve incongruently, and the data points of the aqueous solutions move progressively up to the Lippmann *solutus* curve and then along the *solutus* curve or saturation curve of pure MnCO_3_ to the Mn-poor side. The microcrystalline cores of the spherical crystal aggregates are preferentially dissolved to form core hollows while simultaneously precipitating Mn-rich hexagonal prisms.

**Supplementary Information:**

The online version contains supplementary material available at 10.1186/s12932-021-00075-1.

## Introduction

Manganese (II) is a toxic divalent metal that can be found in excessive amounts in various water bodies, soils and rocks [1, 2]. Its mobility in the natural environment is controlled by various geochemical processes, including its incorporation into carbonates to form rhombohedral calcite-rhodochrosite solid solutions [(Ca,Mn)CO_3_] via a dissolution–recrystallization process [3–6]. Furthermore, the precipitation of solid (Ca, Mn)CO_3_ solutions can be a potential technique to eliminate Mn(II) from wastewater [7]. The study of these solid solutions is therefore valuable to understand the Mn cycle in the environment [8].

Although the ionic radius of Ca^2+^ is approximately 20% larger than that of Mn^2+^, Mn can substitute Ca in the calcite lattice [9]. While the (Ca, Mn)CO_3_-H_2_O system has been broadly investigated in the literature, several essential problems remain unresolved, and many contradictory results have been reported [2]. A limited rhombohedral calcite-rhodochrosite solid solution with a wide miscibility gap can be formed at low temperatures [10, 11]. Conversely, natural calcian rhodochrosite and manganoan calcite have been reported to have a complete component range between calcite and rhodochrosite [4, 12], and a nearly continuous series between CaCO_3_ and MnCO_3_ can be prepared in the laboratory [6, 13].

Despite the importance of authigenic mixed Mn–Ca–Mg carbonates in the geochemical cycle of Mn on the Earth’s surface, very limited studies have been conducted to determine the solubility products and stability domain of (Ca,Mn)CO_3_ solid solutions [4, 6]. The accurate determination of the thermodynamic solubilities of carbonate minerals at low temperatures is therefore essential to the interpretation of the behavior of these minerals in natural waters. Fewer studies have been conducted on solid solutions than on single-component carbonates. Many inconsistent solubility values for MnCO_3_ can be found in the literature. The Gibbs energies of formation (Δ*G*_*f*_°) of MnCO_3_ range from − 809.89 kJ/mol to − 818.81 kJ/mol with solubility products (log_*K*_sp_) from − 9.43 to − 10.99 [5, 14]. Generally, synthetic solids yield lower Δ*G*_*f*_° values and appear to be more soluble than natural solids [5]. In addition, the estimation of the dimensionless Guggenheim coefficient *a*_*0*_ for the (Ca,Mn)CO_3_ solid solutions at low temperature differs greatly. The rhombohedral (Ca,Mn)CO_3_ solid solutions as a complete series has *a*_0_ < 2 (25 °C) [15, 16]. Whereas *a*_*0*_ =  + 3.23 (25 °C) is theoretically calculated [10] and *a*_*0*_ = − 1 ± 3 (20 °C) is derived from the experimental stoichiometric solubilities of synthetic Mn-calcite [Ca_0.75_Mn_0.25_CO_3_] [17]. From the phase relationships in anoxic marine muds, *a*_*0*_ is estimated to be − 3.5 (5 °C) [18], which is nearly equal to the *a*_*0*_ value of − 4 (20 °C) for synthetic Mn-calcite [Ca_0.86_Mn_0.14_CO_3_] [13]. The lack of agreement among (Ca,Mn)CO_3_ solid solutions make it difficult to put forward reliable geochemical models [11].

The dissolution behaviors of the synthesized Ca_0.75_Mn_0.25_CO_3_, Ca_0.52_Mn_0.48_CO_3_ and Ca_0.25_Mn_0.75_CO_3_ in CO_2_-saturated water at 20 °C were studied experimentally, and the dissolution paths were analyzed with respect to the stoichiometric saturation and thermodynamic equilibrium using a Lippmann diagram, showing that the metastable solids equilibrated with aqueous solution through dissolution/crystallization at ambient temperature [4]. During the precipitation of the complete (Ca_1-x_Mn_x_)CO_3_ solid solutions, no Ca^2+^ and Mn^2+^ fractionation between the solid and water was detected at a very high precipitation rate, whereas the Mn^2+^ cations were commonly enriched in the solid phase at a lower precipitation rate [13]. However, the exact formation and stability conditions of rhodochrosite-calcite solid solutions on a mechanistic and quantitative basis are still not fully understood [19]. During the dissolution of four (Ca_1-x_Mn_x_)CO_3_ solids (x = 0–0.12) in acidic solutions, the aqueous Mn^2+^ concentrations first increased and then decreased with the formation of rhodochrosite (MnCO_3_), indicating that the dissolution/precipitation of carbonates might be impacted by impurities in calcite [20]. The synthetic disordered Ca-Mg-rhodochrosite (Mn_0.58_Ca_0.39_Mg_0.03_)CO_3_ dissolved congruently in water at 25 °C, from which the ion activity product (log_IAP) of − 10.39 was extrapolated [6]. In brief, information about the thermodynamic data of (Ca_1-x_Mn_x_)CO_3_ solid solutions is still deficient, although their dissolution/precipitation can exert a significant effect on Mn cycling in the environment.

Therefore, the present work aims to determine the apparent solubility of CaCO_3_–MnCO_3_ solid solutions illustrating some detail of the dissolution process. Firstly, calcite-rhodochrosite solid solutions [(Ca_1-x_Mn_x_)CO_3_] with varying Mn/(Ca + Mn) molar ratios (X_Mn_) from 0.00 to 1.00 are precipitated and characterized. Then, the dissolution process of the obtained solid samples and the release of the components (Mn^2+^, Ca^2+^, CO_3_^2−^/HCO_3_^−^) are experimentally studied by batch tests. Finally, a Lippmann diagram for the (Ca_1-x_Mn_x_)CO_3_ solid solutions is constructed to examine the interaction in the solid solution–aqueous solution (SS-AS) system to evaluate the solubility of Mn-carbonates and the Mn distribution in aqueous environments.

## Experimental methods

### Solid synthesis

Eleven crystal solids (CR-00–CR-10) of the (Ca_1-x_Mn_x_)CO_3_ solid solutions were precipitated by mixing a 2 M Ca + Mn solution with a 0.5 M NH_4_HCO_3_ solution based on following equation: M^2+^ + CO_3_^2−^ = MCO_3_, where M = (Ca + Mn) (Table 1). Analytical reagent grade chemicals and ultrapure water were used. The solids were prepared at room temperature (22 ± 1 °C) by the dropwise addition of 50 mL of a 2 M mixed solution of Ca(NO_3_)_2_ and Mn(NO_3_)_2_ with different Mn/(Ca + Mn) molar ratios into 1000 mL of a vigorously agitated 0.5 M NH_4_HCO_3_ solution in N_2_ gas (Table 1). The resulting suspensions were then stirred for another 10 min. Finally, the precipitates were separated from the solutions by membrane filtration, washed cautiously with ethanol and dried for 24 h at 90 °C.

**Table 1:** Summary of the synthesis and composition of calcite-rhodochrosite solid solutions [(Ca_1-x_Mn_x_)CO_3_]

Sample No	Volume of the precursors (mL)			Mn/(Ca + Mn) molar ratio (x)					
	2 M Ca(NO_3_)_2_	2 M Mn(NO_3_)_2_	0.5 M NH_4_HCO_3_	Starting solution	Synthetic solid^a^	Synthetic solid^b^	After 300d dissolution in water^b^		
							Air-saturated	N_2_-degassed	CO_2_-Saturated
CR-00	50	0	1000	0.00	0.00	0.00	0.00	0.00	0.00
CR-01	45	5	1000	0.10	0.11	0.07	0.12	0.12	0.12
CR-02	40	10	1000	0.20	0.22	0.17	0.24	0.24	0.24
CR-03	35	15	1000	0.30	0.32	0.25	0.35	0.34	0.35
CR-04	30	20	1000	0.40	0.42	0.34	0.46	0.45	0.45
CR-05	25	25	1000	0.50	0.53	0.43	0.56	0.55	0.56
CR-06	20	30	1000	0.60	0.63	0.62	0.66	0.65	0.65
CR-07	15	35	1000	0.70	0.72	0.73	0.74	0.73	0.75
CR-08	10	40	1000	0.80	0.83	0.84	0.84	0.84	0.84
CR-09	5	45	1000	0.90	0.92	0.92	0.93	0.92	0.93
CR-10	0	50	1000	1.00	1.00	1.00	1.00	1.00	1.00

^a^Chemical analysis; ^b^EDS analysis

### Characterization

The components of the obtained solids were estimated by wet chemical analyses, i.e., 10 mg of each solid was decomposed in 20 mL of 1 M nitric acid solution that was then diluted to 100 mL with ultrapure water. The total calcium and manganese contents were determined using inductively coupled plasma-optical emission spectrometry (ICP-OES, Perkin-Elmer Optima 7000DV). All solids were identified crystallographically by comparing their X-ray diffraction (XRD, X’Pert PRO, PANalytical B.V.) patterns with a Cu Kα radiation source at 40 kV and 40 mA to the reference codes 01-081-2027 for calcite and 00-044-1472 for rhodochrosite of the International Center for Diffraction Data (ICDD). Field-emission scanning electron microscopy (FE-SEM, JEOL JEM-7800F) was applied to examine the morphological images of the precipitates and their approximate sizes. The solids were also embedded in resins, polished until their grains had been sectioned and then coated using carbon to study the compositional inhomogeneity and zoning phenomena within the crystals by backscattered electron imaging (BSEI) with the FE-SEM instrument.

### Dissolution experiments

Dissolution experiments of the (Ca_1-x_Mn_x_)CO_3_ solid solutions in a closed system were performed. The working principle of the closed-system was described in detail previously [6]. Instrumental-grade N_2_, air and CO_2_ were used to saturate the initial solutions. Briefly, 5 g of the (Ca_1-x_Mn_x_)CO_3_ solid solutions were weighed and added into three groups of labeled 100 mL polyethylene terephthalate (PET) bottles, and 100 mL of N_2_-degassed, air-saturated and CO_2_-saturated ultrapure water was then used to fill the bottles. After being capped, the bottles were immersed in water baths at 25 °C. At various intervals (1, 3, 6, 12, 24, 48, 72, 120, 240, 360, 480, 720, 960, 1200, 1440, 1680, 1920, 2160, 2400, 4800 and 7200 h) after the start of the experiment, one bottle from each group was removed. The pH values were immediately measured, and the solutions were filtered with 0.45 µm membrane filters. The HCO_3_^−^/CO_3_^2-^ concentrations were measured by using an automatic potentiometric titrator (888 Titrando, Metrohm, Switzerland). Additionally, 5 mL of the solutions was filtered through 0.22 µm membranes and acidified in 25 mL flasks using 0.2% nitric acid. The total calcium and manganese contents were determined using ICP-OES. At the experimental end, the solid residuals were collected from the bottles, washed with ethanol, dried at 90 °C for 24 h, and characterized using XRD and FE-SEM as previously described.

### Thermodynamic calculations

For the dissolution of the (Ca_1-x_Mn_x_)CO_3_ solid solutions, the activities of free Mn^2+^, Ca^2+^ and CO_3_^2−^ were first estimated using the geochemical code PHREEQC (Version 3.6.2) [21], and then the ion activity products (IAPs) were calculated after the definitions, which equaled the solubility products (*K*_sp_) of the (Ca_1-x_Mn_x_)CO_3_ solid solutions at equilibrium. The component species involved in the simulation contained Mn^2+^, MnOH^+^, Mn(OH)_2_^0^, Mn(OH)_3_^−^, Mn(OH)_4_^2−^ and MnHCO_3_^+^ for manganese; Ca^2+^, CaOH^+^, CaHCO_3_^+^ and CaCO_3_^0^ for calcium; and CO_3_^2−^, HCO_3_^−^, H_2_CO_3_^0^, CaHCO_3_^+^ and MnHCO_3_^+^ for carbonate. The built-in database minteq.v4.dat of PHREEQC included the thermodynamic properties of all solid phases and aqueous species for modeling (Additional file 1: Appendix S1). The ionic strength values (< 0.01166 mol/L) lay in the applicable range for the extended Debye-Hückel equation.

## Results and discussion

### Solid characterization

#### Composition

The compositions of the obtained solids were close to the Mn/(Ca + Mn) mole ratios of the starting solution (Table 1). The predicted Mn/(Ca + Mn) molar ratios (X_Mn_) were not linearly correlated with the measured X_Mn_, showing the preferential Mn uptake of the solids [5]. After the dissolution of the (Ca_1-x_Mn_x_)CO_3_ solid solutions in the N_2_-degassed, air-saturated and CO_2_-saturated water samples for 300 d, the Mn/(Ca + Mn) molar ratios (X_Mn_) of the residual solids increased slightly, indicating the preferential retention of Mn in the solids.

#### XRD

**Fig. 1:**
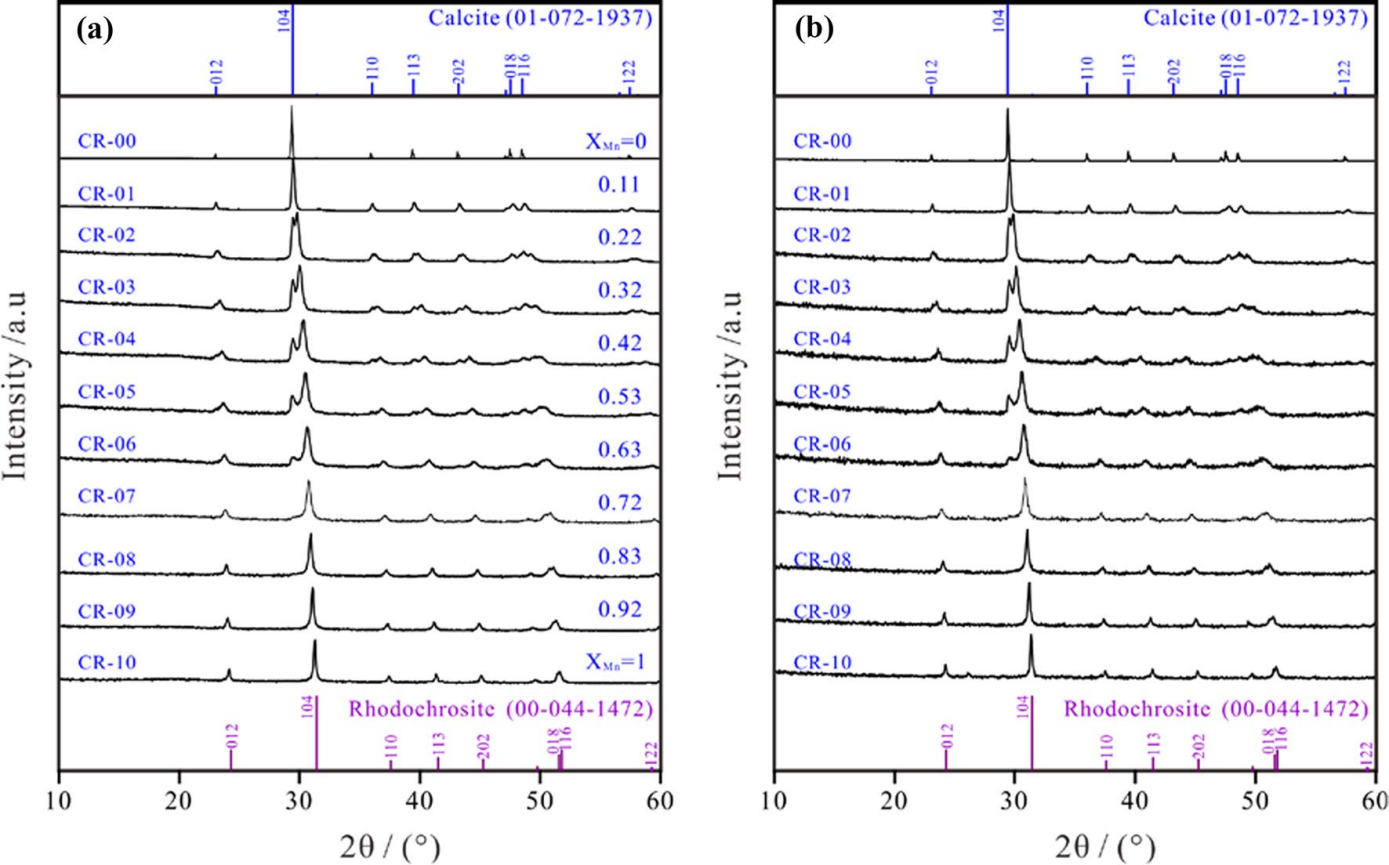
Diffraction patterns of the (Ca_1-x_Mn_x_)CO_3_ solid solutions (**a**) before and (**b**) after dissolution in air-saturated water for 300 d

The synthesized crystals belonged to calcite–rhodochrosite [(Ca_1-x_Mn_x_)CO_3_] solid solutions, and all XRD spectra were confirmed to be the *R* $\overline{3}$ *c* space group (Fig. 1). The crystals of X_Mn_ = 0 and 1 (CR-00 and CR-10) were identical to calcite (ICSD Reference code 01-072-1937) and rhodochrosite (00-044-1472), respectively. The reflection peaks, particularly (104), shifted slightly to higher angles with increased Mn incorporation, owing to the smaller interplanar distance of Mn-calcite in comparison with pure calcite. All well-crystallized solids differed only in the peak locations, widths and intensities in their XRD spectra, indicating that they were not simple mixtures of the calcite and rhodochrosite endmembers but rather calcite–rhodochrosite [(Ca_1-x_Mn_x_)CO_3_] solid solutions (Fig. 1; Additional file 1: Appendix S2-A **)**.

Regarding the solids that were formed from aqueous solutions with low Mn^2+^/Ca^2+^ ratios (CR-00 and CR-01) or high Mn^2+^/Ca^2+^ ratios (CR-07–CR-10), only the peaks corresponding to the calcite or rhodochrosite endmembers were found in the XRD patterns (Fig. 1). Regarding the solids that were formed from aqueous solutions with intermediate Mn^2+^/Ca^2+^ ratios (CR-02–CR-06), all the XRD patterns exhibited double peaks, and as a result, two groups of reflections were considered for indexing: in the first group, the 2θ angles were close to the corresponding reflections of calcite and lower than the second group whose reflections were closer to the rhodochrosite peaks. The presence of double peaks indicated compositional heterogeneities in the samples, which was also confirmed in the electron microscopy observations and microprobe analyses. As the Mn^2+^/Ca^2+^ ratios in the starting solutions increased, the reflections assigned to the Mn-rich zones became progressively more intense [2].

Additional reflections that could be attributed to ordered kutnohorite were not found in any of the XRD patterns [22]. After dissolution for 300 d, no obvious variations, including additional peaks for portlandite [Ca(OH)_2_] and pyrochroite [Mn(OH)_2_], were found for all solids (Fig. 1; Additional file 1: Appendix S2-B **)**.

#### SEM

The morphologies of the crystals exhibited a strong dependence on the Mn concentrations in the starting solutions. A variety of crystal morphologies were obtained, showing the effects of Mn^2+^ (even at low concentrations) on the growth of the {104} faces of calcite [2, 11].

**Fig. 2:**
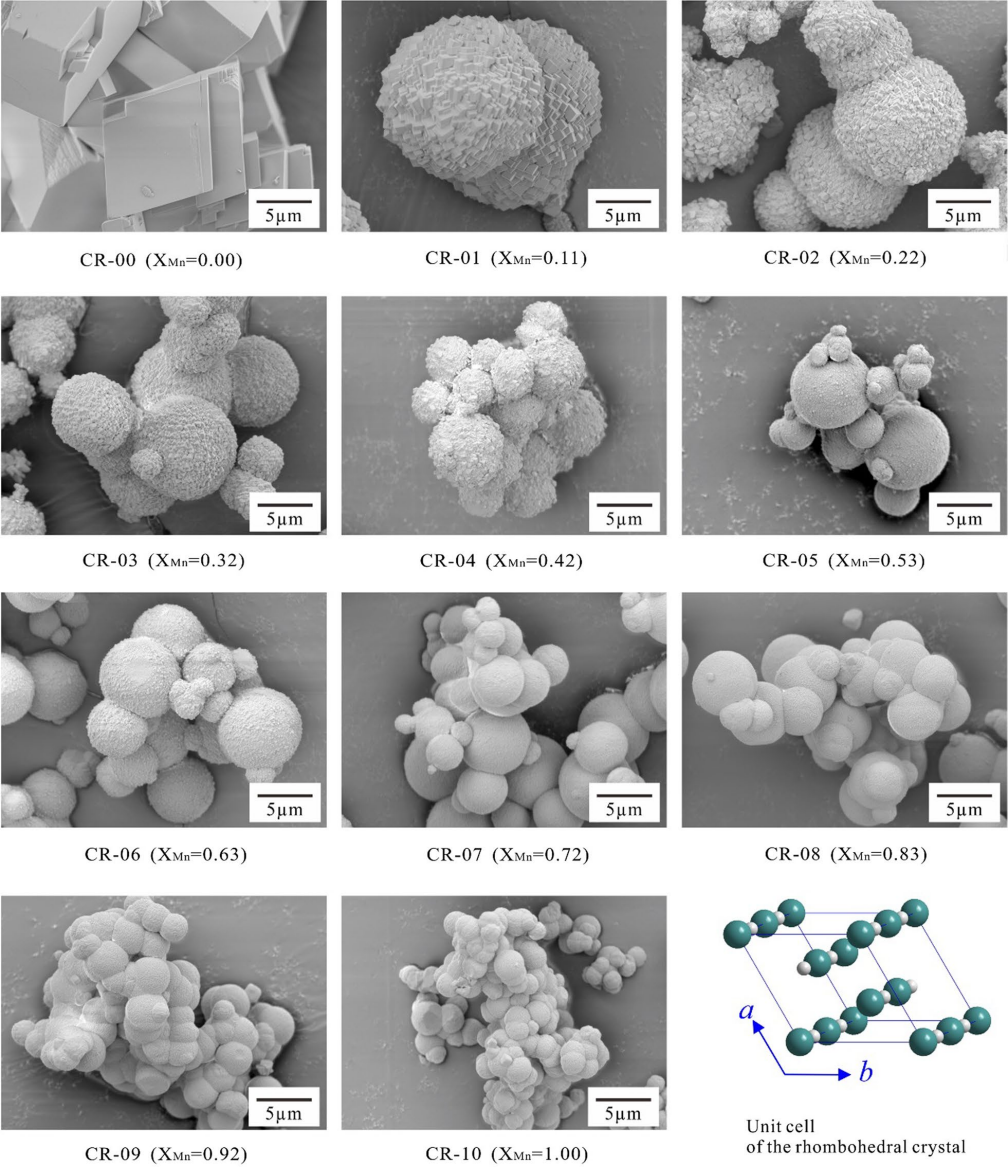
SEM images of the (Ca_1-x_Mn_x_)CO_3_ solid solutions before dissolution

The synthesized calcite (CR-00) showed a typical morphology with rhombohedral habits defined by {104} planes with a grain size of ~ 10 µm (Fig. 2; Additional file 1: Appendix S3). The sizes of the (Ca_1-x_Mn_x_)CO_3_ solid solutions decreased with an increasing X_Mn_, although the morphologies remained rhombohedral for the (Ca_1-x_Mn_x_)CO_3_ aggregations (Fig. 2), showing that manganese could influence the growth and morphology of calcite [11, 20]. As the Mn concentrations increased, the (Ca_1-x_Mn_x_)CO_3_ solid solutions varied from blocky crystals to spherulites and peanut-like aggregates [23]. The crystal morphologies of the intermediate compositions and the MnCO_3_ endmember (X_Mn_ = 0.11–1.00; CR-01–CR-10) varied from blocky spherical crystal aggregates to smaller spheres following the change in the Mn contents of the initial solutions (Fig. 2). The individual was a crystal, the surface of which was formed by the aggregation of numerous minuscule blocks of {104} facets that appeared slightly disoriented, as they traced the external shape of a solid that could be considered spherical. This final morphology was a result of intense splitting phenomena during the growth process [2].

The increase in the relative amounts of Mn in the starting solution could cause a further modification of the external morphology of the crystals (Fig. 2; Additional file 1: Appendix S3). With increasing Mn concentrations, the size of the individual crystals decreased, and the terrace texture of the external morphology was eliminated and replaced by a very smooth and rounded surface. This morphological modification was also accompanied by a decrease in the ‘physical’ spherical particle diameters. This decrease in crystal sizes was expected due to the lower solubilities of Mn-rich carbonates in comparison to Ca-rich carbonates, i.e., the less soluble Mn-rich phases were more metastable; consequently, they crystallized at a higher supersaturation [24]. This higher supersaturation caused a larger nucleation density, consequently decreasing the crystal size. Similar results have also been observed in earlier studies [2, 23].

**Fig. 3:**
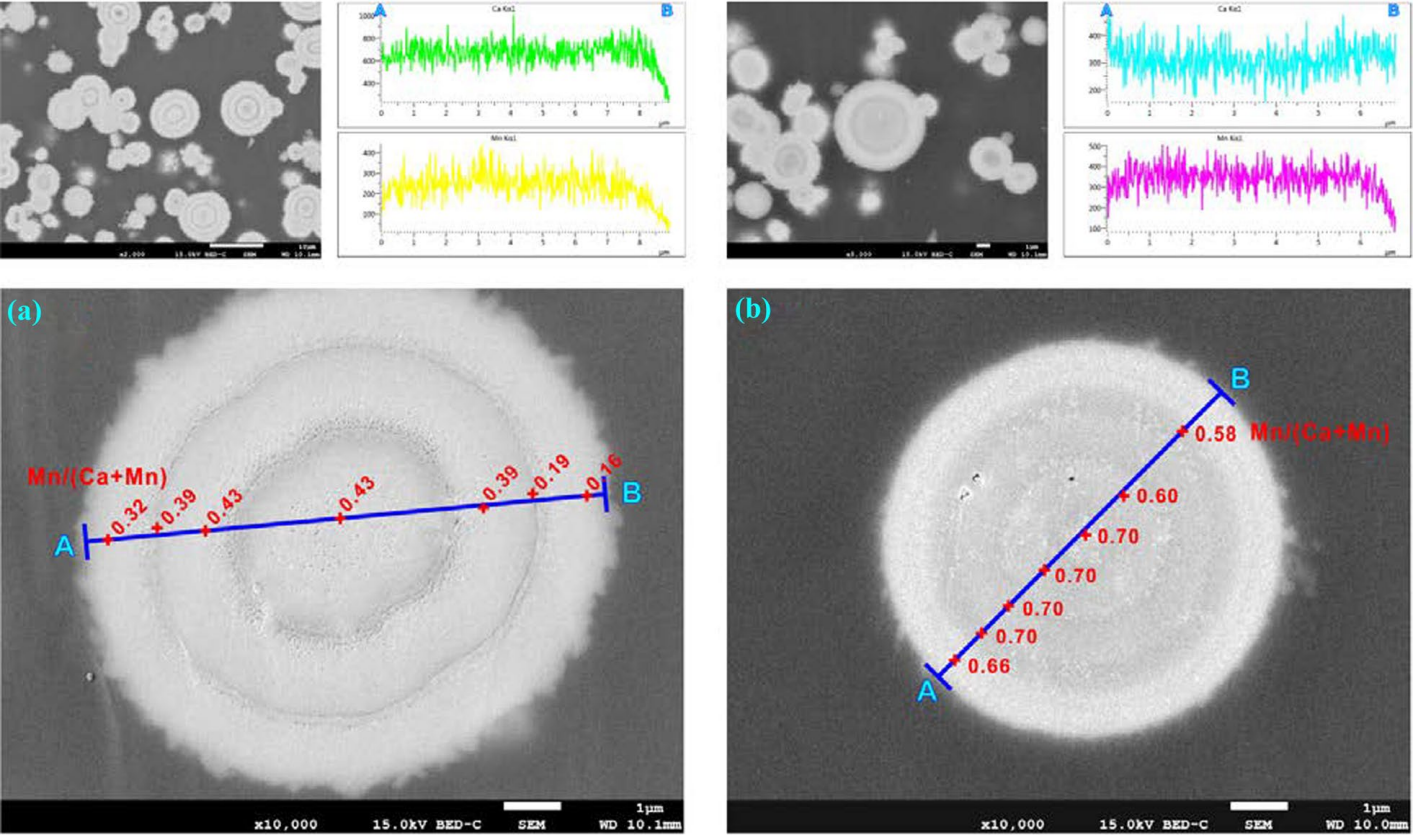
BSE images of the equatorial sections and the corresponding compositional profiles along the A-B line of the (**a**) (Ca_0.68_Mn_0.32_)CO_3_ (CR-03) and (**b**) (Ca_0.48_Mn_0.52_)CO_3_ (CR-05) solids before dissolution

**Fig. 4:**
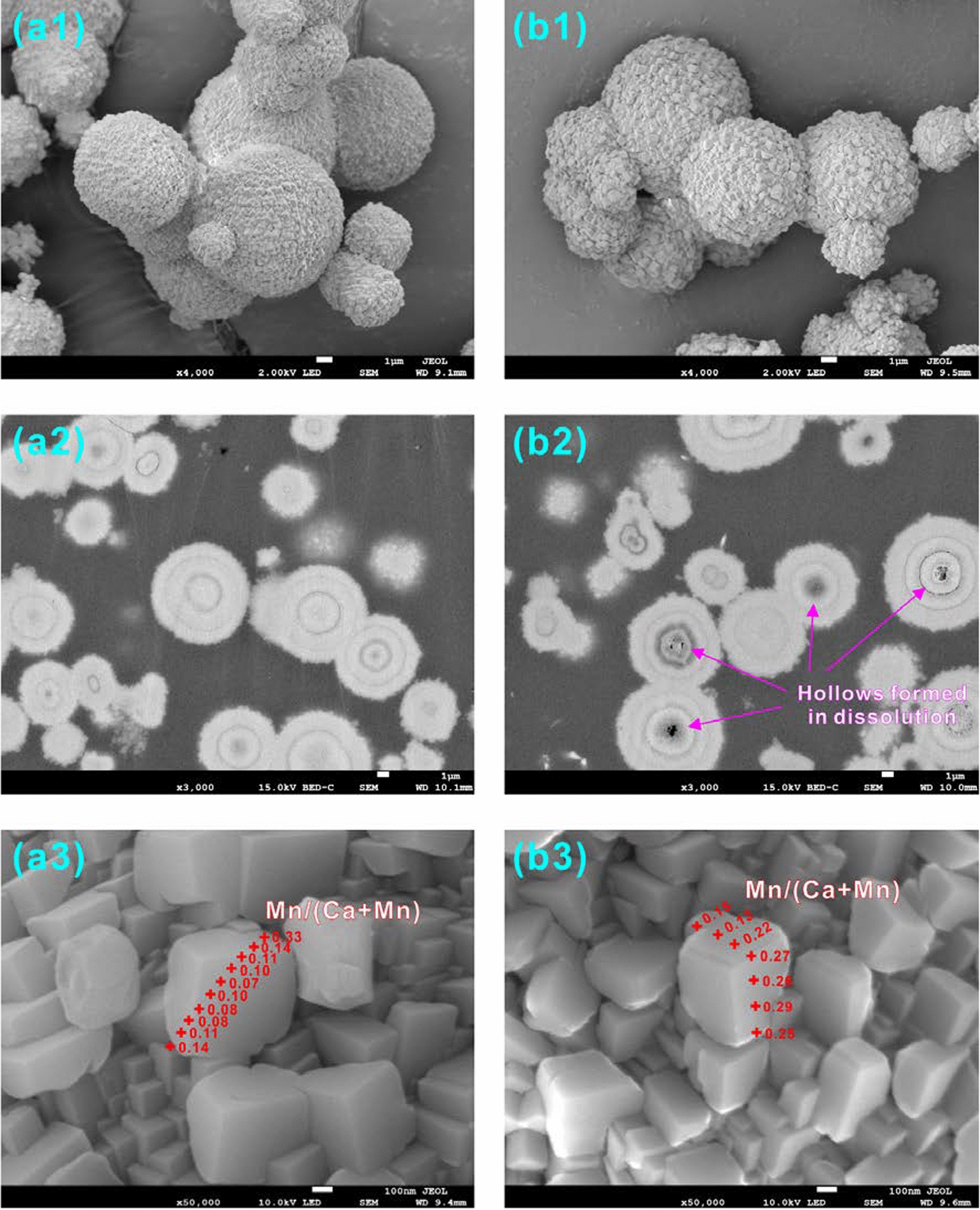
SEM and BSE images of (Ca_0.68_Mn_0.32_)CO_3_ (CR-03) solid (**a**) before and (**b**) after dissolution in air-saturated water for 300 d

**Fig. 5:**
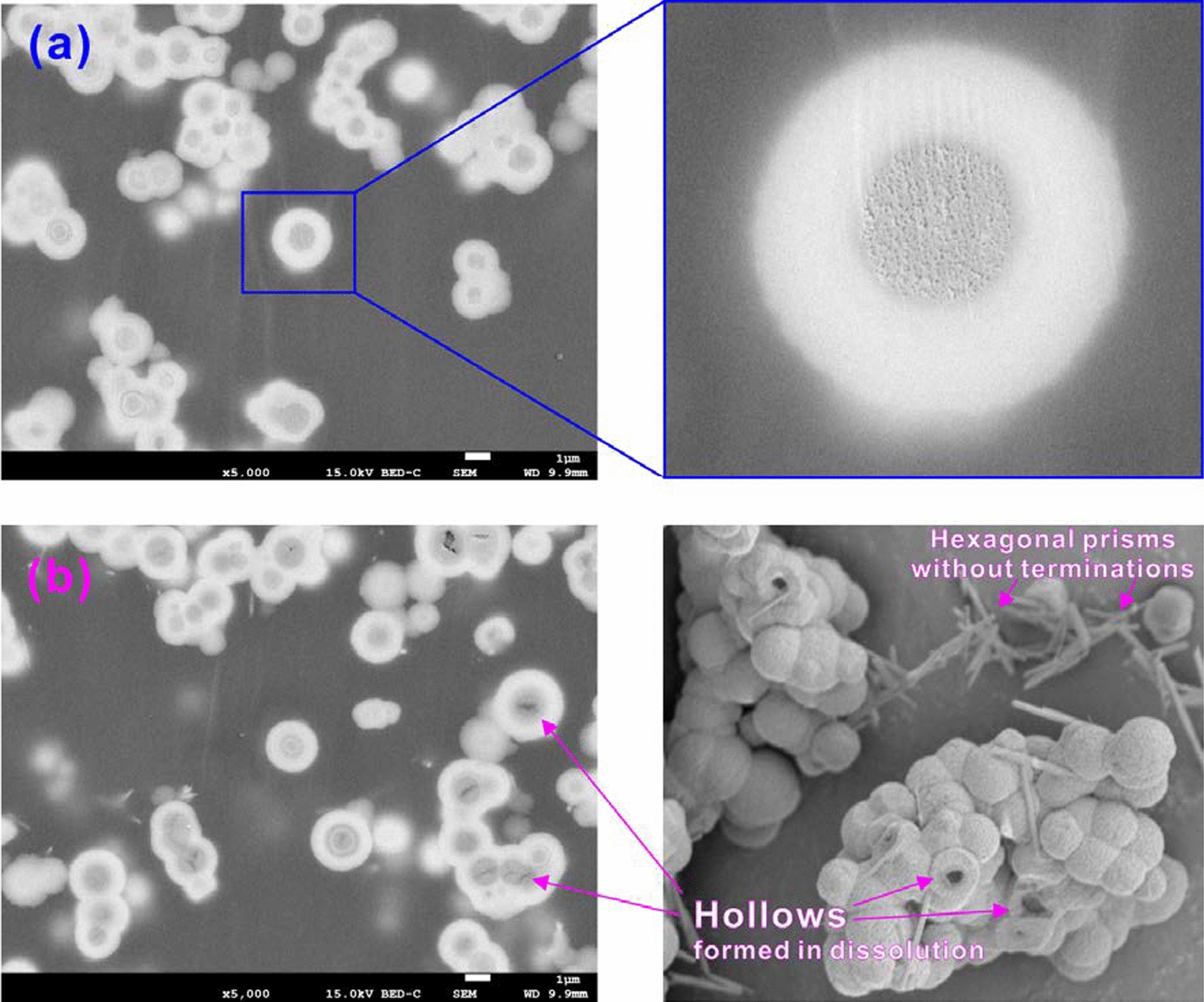
SEM and BSE images of pure MnCO_3_ (CR-10) (**a**) before and (**b**) after dissolution in air-saturated water for 300 d to show the formation of hollow spheres and hexagonal prisms without terminations

Sections of the crystal aggregates (CR-01–CR-10) grown from starting aqueous solutions containing Mn^2+^ showed clear oscillatory concentric zoning, which indicated the core-to-rim compositional heterogeneity of the bulk crystal spheres (Figs. 3 and 4; Additional file 1: Appendix S4 and S5) or the core-to-rim crystalline heterogeneity of the pure MnCO_3_ spheres (CR-10) (Fig. 5). Since variations in image brightness represented changes in composition, the visibility of compositional zoning could be attained by the backscattered imaging of polished central sections of crystals. Backscattered electron images of some representative individuals (CR-03 and CR-05) together with the corresponding compositional profiles are shown in Fig. 3 before dissolution and in Additional file 1: Appendix S4 after dissolution. The central part of the crystal was always relatively rich in Mn. Surrounding this core were successive Ca-rich and Mn-rich rings [2]. The thickness of these zones ranged from approximately 0.8 µm, and variations within a ring never exceeded an X_Mn_ value of 0.10. In the outer part, the morphology of an initial rhombohedron could still be seen (Additional file 1: Appendix S5).

High compositional gradients of crystals have been proven to be a major characteristic of solid solutions with a great difference in the solubilities of the endmembers (2 to 3 orders of magnitude) [25, 26] and could be easy to explain considering changes in the fluid composition during crystal growth [2, 27, 28]. The Mn-rich phases of the (Ca,Mn)CO_3_ solid solutions were clearly much less soluble (~ 2 orders of magnitude) than the Ca-rich phases, so Mn was preferentially incorporated in the solid phases at the primary nucleation stage. The consumption of Mn in the bulk crystals caused the local depletion of Mn^2+^ in the solutions and the subsequent growth of Ca-rich solid phases. The Ca incorporation in the crystal reduced the Ca concentrations in the nucleation area, and Mn once again started to be preferentially incorporated toward the solids. Because of the large difference in the solubilities of calcite and rhodochrosite, a very small change in the solution composition during Mn enrichment or depletion could cause a large compositional gradient in the growing solid [2].

Generally, all solids after dissolution showed the same macroscopic characteristics as before, i.e., the endmember calcite (CR-00) had a common appearance with rhombohedral habits, and the morphologies of crystals of the intermediate compositions and the endmember rhodochrosite (CR-01–CR-10) changed from blocky spherical crystal aggregates to spheres following the increase in Mn content (Fig. 2; Additional file 1: Appendix S3).

After calcite dissolution, the edges of the crystals degenerated, and etch pits on the surface were observed (Additional file 1: Appendix S3). Furthermore, it was observed that the microcrystalline cores of the spherical crystal aggregates (CR-01–CR-10) were preferentially dissolved to form hollows while simultaneously precipitating Mn-rich hexagonal prisms (Figs. 4 and  5; Additional file 1: Appendix S5). The rhombohedral crystals were not fully closed so solution could flow into the core of the sphere, which resulted in the first dissolution of the core crystals that had a smaller grain size than the peripheral crystals. Needle-like crystals or hexagonal prisms without rhombohedral terminations more clearly formed with an increasing dissolution time and Mn/(Ca + Mn) molar ratio (X_Mn_) during the dissolution of solids containing Mn (CR-01–CR-10). EDS analyses indicated that the acicular crystals or hexagonal prisms clearly had a higher Mn content than the bulk composition of the original solid (Additional file 1: Appendix S5). The rhodochrosite precipitate was monocrystalline [20, 29]. It was also observed that some rod-like phases formed during the interaction between the calcite crystals and Mn^2+^ solution [30, 31].

### Variation of the aqueous solution

**Fig. 6:**
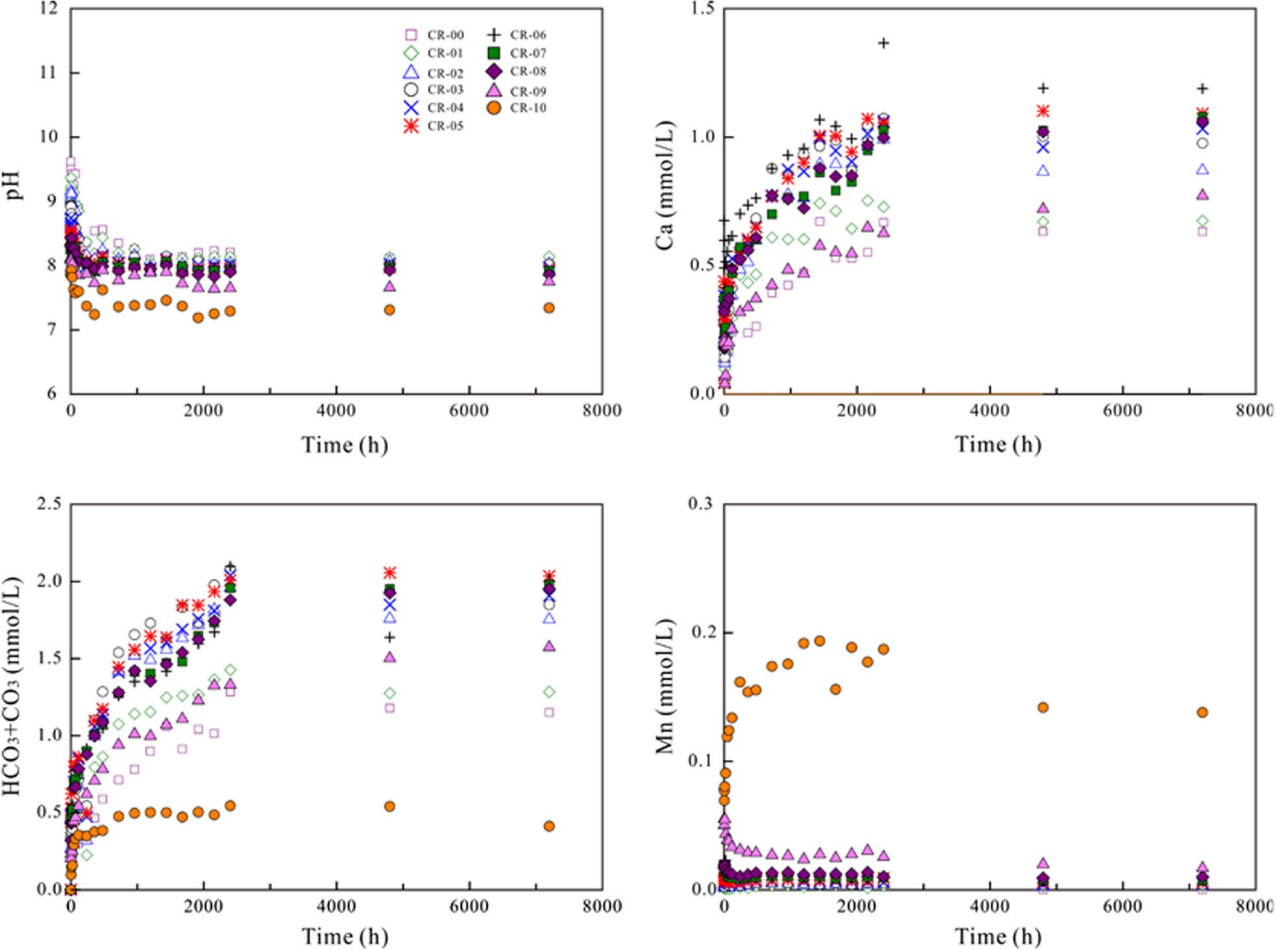
Variation in the aqueous pH and components during the dissolution of the (Ca_1-x_Mn_x_)CO_3_ solid solutions in air-saturated water

**Fig. 7:**
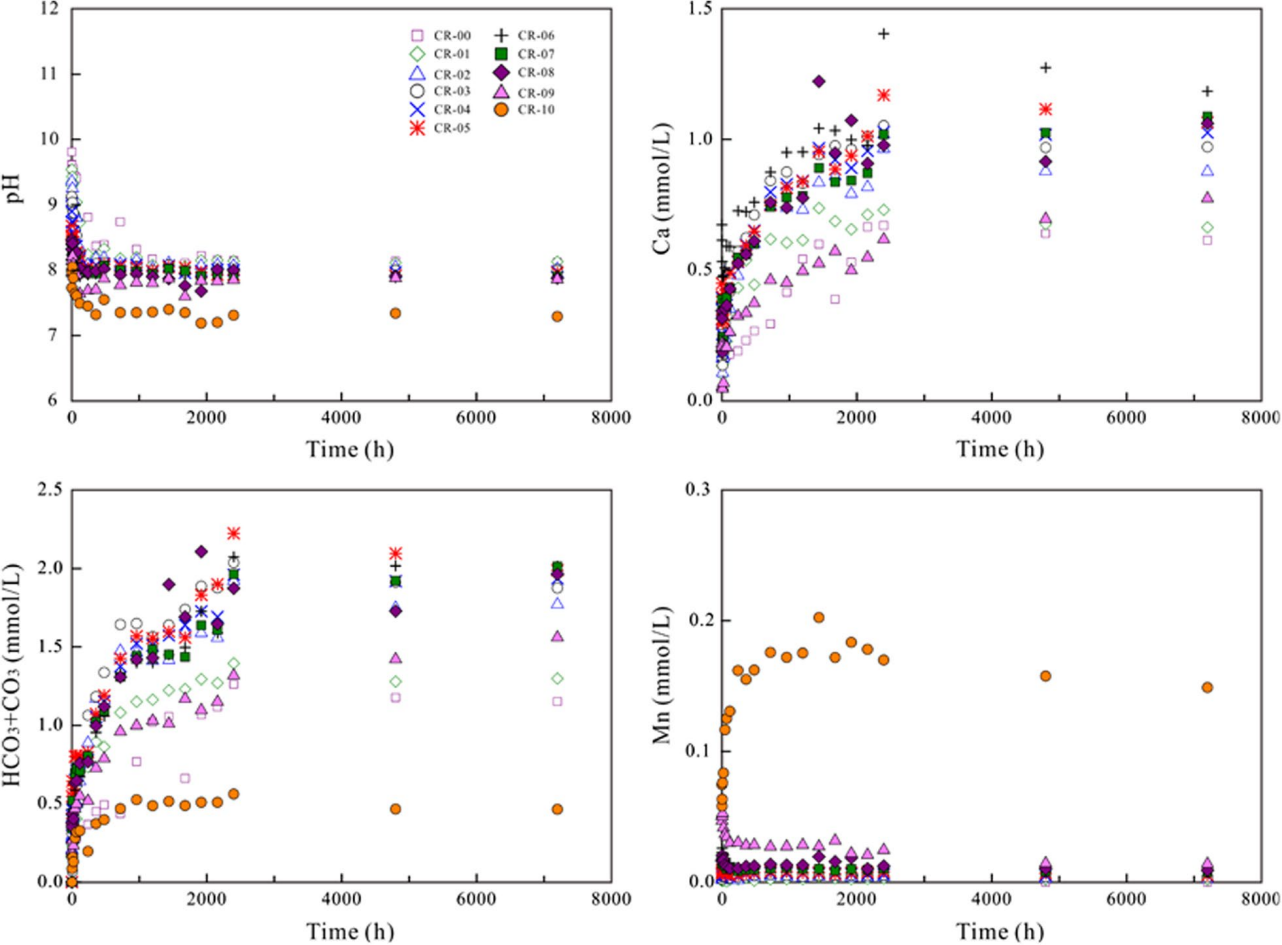
Variation in the aqueous pH and components during the dissolution of the (Ca_1-x_Mn_x_)CO_3_ solid solutions in N_2_-degassed water

Regarding dissolution in air-saturated water and N_2_-degassed ultrapure water, the aqueous pH decreased gradually to a steady state (Figs. 6 and  7). The aqueous Ca and HCO_3_ + CO_3_ concentrations increased up to the highest value after 1240–2400 h of dissolution for all (Ca,Mn)CO_3_ solid solutions. The aqueous Mn concentrations increased up to the highest value of ~ 0.20 mmol/L after 1440 h and then decreased gradually to a steady state for pure rhodochrosite [MnCO_3_] (CR-10). Regarding the Mn-containing solids (CR-01–CR-09), the aqueous Mn concentrations and the aqueous Mn/(Ca + Mn) mole ratios increased up to the highest value after 6–12 h of dissolution and then decreased gradually to a steady state (Additional file 1: Appendix S6). The X-ray photoelectron spectroscopy (XPS) analysis showed that Mn^2+^ ions were not oxidized during the dissolution in air-saturated initial solutions for the closed-system experiments (Additional file 1: Appendix S7).

**Fig. 8:**
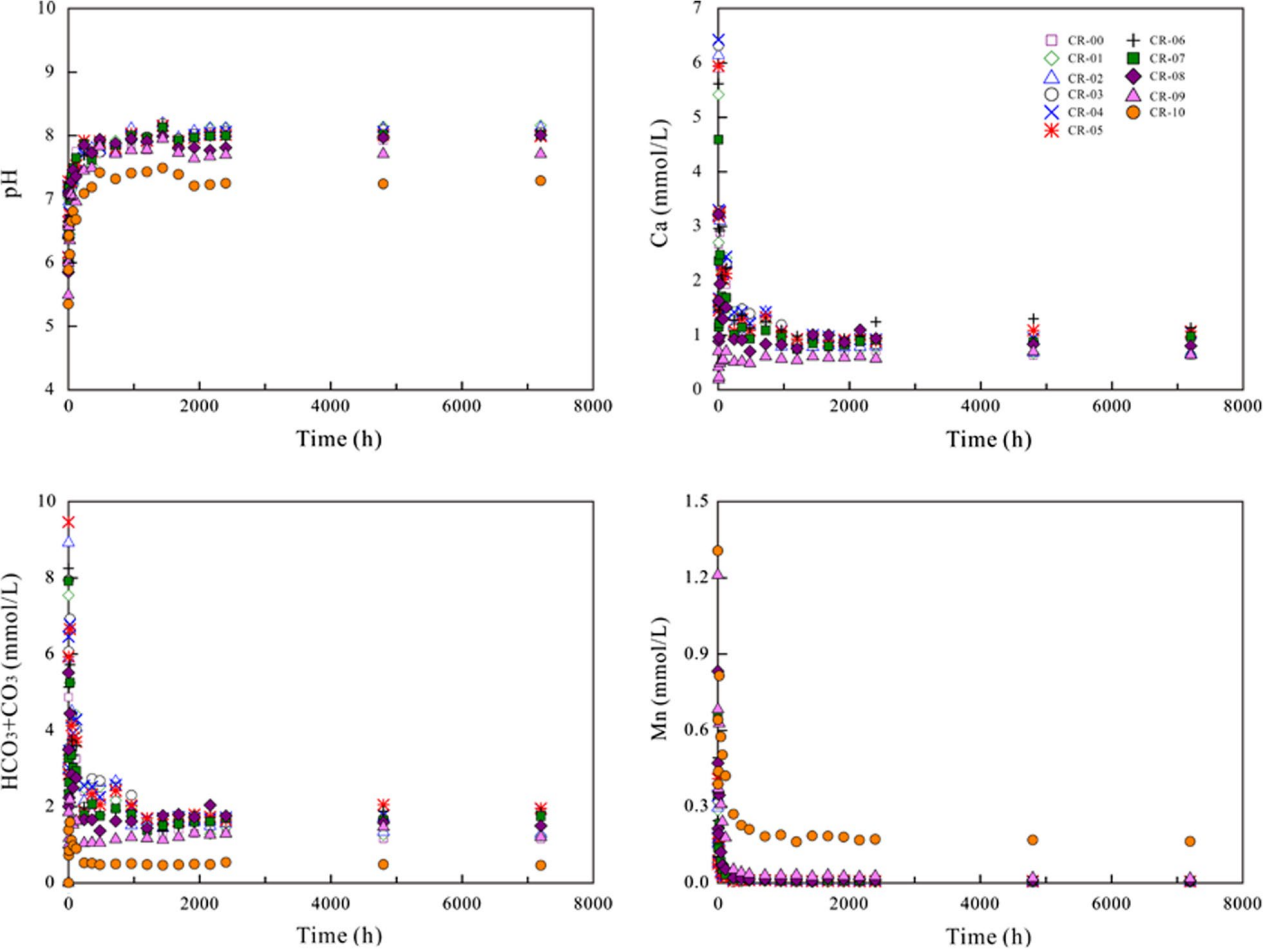
Variation in the aqueous pH and components during the dissolution of the (Ca_1-x_Mn_x_)CO_3_ solid solutions in CO_2_-saturated water

Regarding dissolution in CO_2_-saturated water, the aqueous pH increased rapidly from 4.26 of the starting solutions to 5.35–6.06 within 1 h of dissolution and then gradually reached a steady state (Fig. 8). Generally, the aqueous concentrations of Ca, Mn and HCO_3_ + CO_3_ reached maximum values within 1 h of dissolution and then decreased to steady states with a slight fluctuation between 6 and 24 h. The aqueous Mn/(Ca + Mn) mole ratios increased up to the highest value after 1–6 h of dissolution and then decreased gradually to a steady state (Additional file 1: Appendix S6).

A large amount of the released Mn was eliminated from solutions, probably through reprecipitation/recrystallization. However, the Mn/(Ca + Mn) molar ratios in solution were clearly lower than X_Mn_ of the corresponding solid, indicating a nonstoichiometric release of Mn and Ca (Additional file 1: Appendix S6 and S8). It is also worth noting that the aqueous pH decreased and the aqueous Mn concentrations increased with an increasing X_Mn_ of the (Ca,Mn)CO_3_ solid solutions. The aqueous Ca and HCO_3_ + CO_3_ concentrations showed the highest values at X_Mn_ = 0.53–0.63 (CR-05–CR-06) and then decreased as the X_Mn_ values decreased or increased (Additional file 1: Appendix S8).

The stoichiometric dissolution of the (Ca_1-x_Mn_x_)CO_3_ solid solutions is as follows:

1 $$\left( {{\text{C}}{{\text{a}}_{1 - {\text{X}}}}{\text{M}}{{\text{n}}_{\text{X}}}} \right){\text{C}}{{\text{O}}_{\text{3}}} = \left( {1 - {\text{X}}} \right){\text{Ca}}_{\left( {{\text{aq}}} \right)}^{2 + } + {\text{XMn}}_{\left( {{\text{aq}}} \right)}^{2 + } + {\text{CO}}_{3\left( {{\text{aq}}} \right)}^{2 - }$$

2 $${\text{Ca}}^{{{2} + }} + {\text{ OH}}^{ - } = {\text{Ca}}\left( {{\text{OH}}} \right)^{ + }$$

3 $${\text{Mn}}^{{{2} + }} + {\text{ nOH}}^{ - } = {\text{ Mn}}\left( {{\text{OH}}} \right)_{{\text{n}}}^{{\left( {{\text{n}} - {2}} \right) - }} \quad \left( {{\text{n}} = {1} \sim {4}} \right)$$

4 $${\text{Ca}}^{{{2} + }} + {\text{ H}}^{ + } + {\text{ CO}}_{{3}}^{{{2} - }} = {\text{ CaHCO}}_{{3}}^{ + }$$

5 $${\text{Mn}}^{{{2} + }} + {\text{ H}}^{ + } + {\text{ CO}}_{{3}}^{{{2} - }} = {\text{ MnHCO}}_{{3}}^{ + }$$

6 $${\text{CO}}_{{3}}^{{{2} - }} + {\text{ nH}}^{ + } = {\text{H}}_{{\text{n}}} {\text{CO}}_{{3}}^{{\left( {{2} - {\text{n}}} \right) - }} \quad \left( {{\text{n}} = {1},{ 2}} \right)$$

At the early stage, all components were dissolved stoichiometrically (Eq. (1)). Regarding the dissolution in N_2_-degassed water and air-saturated water, the pH decrease was ascribed to the formation of Ca(OH)^+^ and Mn(OH)_n_^(n−2)−^ (Eqs. (2) and (3)), which resulted in OH^−^ depletion. Regarding the dissolution in CO_2_-saturated water, the pH increase was ascribed to the formation of CaHCO_3_^+^, MnHCO_3_^+^ and H_n_CO_3_^(2−n)−^ (Eqs. (4)–(6)), which resulted in H^+^ depletion. The notable pH variation showed that the initial dissolution was pH-controlled [20, 32]. More calcium was dissolved than manganese, indicating that the (Ca_1-x_Mn_x_)CO_3_ solid solutions dissolved nonstoichiometrically [20] and suggesting the existence of an interfacial dissolution–precipitation process; thus, manganese more favorably nucleated on the crystal surfaces to form new precipitates [20, 33].

### Determination of the stoichiometric solubility

Dissolution was carried out until the differences in the calculated IAP values for the final three solutions (i.e., 2400 h, 4800 h and 7200 h; assuming a steady state was reached) were mostly within ± 0.25 log units [34]. The PHREEQC simulation results indicated that all aqueous solutions in the present research were undersaturated with respect to any probable secondary mineral phases, including portlandite [Ca(OH)_2_, saturation indexes (SI) = − 6.68 ~ − 15.10] and pyrochroite [Mn(OH)_2_, SI = − 1.27 ~ − 14.98].

The thermodynamic solubility product could be calculated from the long-term steady states or extrapolated ion activity products (IAPs) of the solutions that corresponded to the equilibrium constant of mineral dissolution [6]. The equilibrium constant (*K*_sp_, the stoichiometric solubility product) of the dissolution after Eq. (1) at dissolution equilibrium can be estimated by Eq. (7):

7 $$K_{{{\text{sp}}}} = {\text{ IAP }} = {\mkern 1mu} \left\{ {{\text{Ca}}^{{2 + }} } \right\}^{{1 - {\text{x}}}} \left\{ {{\text{Mn}}^{{2 + }} } \right\}^{{\text{x}}} \left\{ {{\text{CO}}_{3}^{{2 - }} } \right\}$$

where {} is the free ion activity.

The standard free energy of reaction (Δ*G*_*r*_^*o*^) can be calculated from *K*_sp_ at 298.15 K and 0.101 MPa with Eq. (8):

8 $$\Delta G_{r}^\circ \, = \, - {5}.{7}0{\text{8 log}}\_K_{{{\text{sp}}}}$$

For Eq. (1),

9 $$\Delta G_{r}^\circ \, = \, \left( {{1} - {\text{x}}} \right)\Delta G_{f}^\circ \left[ {{\text{Ca}}^{{{2} + }} } \right] \, + {\text{ x}}\Delta G_{f}^\circ \left[ {{\text{Mn}}^{{{2} + }} } \right] \, + \Delta G_{f}^\circ \left[ {{\text{CO}}_{{3}}^{{{2} - }} } \right] \, - \Delta G_{f}^\circ \left[ {\left( {{\text{Ca}}_{{{1} - {\text{x}}}} {\text{Mn}}_{{\text{x}}} } \right){\text{CO}}_{{3}} } \right]$$

Rearranging, we obtain:

10 $$\Delta G_{f}^\circ \left[ {\left( {{\text{Ca}}_{{{1} - {\text{x}}}} {\text{Mn}}_{{\text{x}}} } \right){\text{CO}}_{{3}} } \right] \, = \, \left( {{1} - {\text{x}}} \right)\Delta G_{f}^\circ \left[ {{\text{Ca}}^{{{2} + }} } \right] \, + {\text{ x}}\Delta G_{f}^\circ \left[ {{\text{Mn}}^{{{2} + }} } \right] \, + \, \Delta G_{f}^\circ \left[ {{\text{CO}}_{{3}}^{{{2} - }} } \right] \, - \Delta G_{r}^\circ$$

Tables 2, 3 and 4 list the pH, Ca, Mn and HCO_3_^−^/CO_3_^2−^ analytical results and the calculated log_IAP values at the final steady state (≈log_*K*_sp_) for the (Ca_1-x_Mn_x_)CO_3_ solid solutions. Based on the obtained literature data, Δ*G*_*f*_°[Ca^2+^] = − 553.54 kJ/mol, Δ*G*_*f*_°[Mn^2+^] = − 228 kJ/mol and Δ*G*_*f*_°[CO_3_^2−^] = − 527.9 kJ/mol [35], the free energies of formation, Δ*G*_*f*_°[(Ca_1-x_Mn_x_)CO_3_], were also estimated.

**Table 2:** Analytical data and solubility of the (Ca_1-x_Mn_x_)CO_3_ solid solutions in air-saturated water at 25 °C

Sample	Dissolution time (h)	pH	Concentration (mmol/L)			log_IAP^c^	Average log_IAP	ΔG_*f*_° (kJ/mol)	Average ΔG_*f*_° (kJ/mol)
			Ca	Mn	HCO_3_ + CO_3_				
(Ca_1.00_Mn_0.00_)CO_3_	2400	8.21	0.6677	0.0000	1.2831	− 8.34	− 8.44	− 1129.04	− 1129.63
	4800	8.12	0.6330	0.0000	1.1790	− 8.48	± 0.10	− 1129.87	± 0.59
	7200	8.11	0.6323	0.0000	1.1507	− 8.50		− 1129.98	
(Ca_0.89_Mn_0.11_)CO_3_^a^	2400	8.14	0.7281	0.0031	1.4260	− 8.62	− 8.68	− 1094.68	− 1095.02
(Ca_0.88_Mn_0.12_)CO_3_^b^	4800	8.13	0.6702	0.0023	1.2751	− 8.72	± 0.06	− 1095.24	± 0.34
	7200	8.14	0.6749	0.0025	1.2841	− 8.70		− 1095.13	
(Ca_0.78_Mn_0.22_)CO_3_^a^	2400	8.04	0.9911	0.0046	1.9561	− 8.74	− 8.83	− 1059.46	− 1059.92
(Ca_0.76_Mn_0.24_)CO_3_^b^	4800	8.07	0.8657	0.0026	1.7596	− 8.85	± 0.08	− 1060.06	± 0.46
	7200	8.03	0.8708	0.0030	1.7536	− 8.88		− 1060.22	
(Ca_0.68_Mn_0.32_)CO_3_^a^	2400	7.99	1.0729	0.0052	2.0771	− 8.99	− 9.04	− 1028.19	− 1028.46
(Ca_0.65_Mn_0.35_)CO_3_^b^	4800	8.01	1.0029	0.0039	1.9388	− 9.06	± 0.05	− 1028.55	± 0.27
	7200	8.02	0.9766	0.0039	1.8492	− 9.07		− 1028.64	
(Ca_0.58_Mn_0.42_)CO_3_^a^	2400	8.01	1.0624	0.0057	2.0395	− 9.21	− 9.28	− 996.79	− 997.16
(Ca_0.54_Mn_0.46_)CO_3_^b^	4800	8.02	0.9609	0.0043	1.8495	− 9.32	± 0.07	− 997.37	± 0.37
	7200	7.98	1.0321	0.0049	1.9072	− 9.31		− 997.31	
(Ca_0.47_Mn_0.53_)CO_3_^a^	2400	7.96	1.0554	0.0091	2.0089	− 9.39	− 9.45	− 962.13	− 962.49
(Ca_0.44_Mn_0.56_)CO_3_^b^	4800	7.97	1.1016	0.0058	2.0568	− 9.47	± 0.07	− 962.57	± 0.36
	7200	7.94	1.0914	0.0058	2.0347	− 9.51		− 962.78	
(Ca_0.37_Mn_0.63_)CO_3_^a^	2400	8.00	1.4088	0.0100	2.0973	− 9.48	− 9.62	− 930.11	− 930.87
(Ca_0.34_Mn_0.66_)CO_3_^b^	4800	8.01	1.1903	0.0062	1.6380	− 9.73	± 0.13	− 931.48	± 0.76
	7200	7.96	1.1889	0.0073	2.0109	− 9.64		− 931.02	
(Ca_0.28_Mn_0.72_)CO_3_^a^	2400	7.93	1.0275	0.0095	1.9579	− 9.79	− 9.82	− 902.68	− 902.89
(Ca_0.26_Mn_0.74_)CO_3_^b^	4800	7.98	1.0251	0.0074	1.9515	− 9.82	± 0.05	− 902.84	± 0.26
	7200	7.92	1.0789	0.0074	1.9774	− 9.87		− 903.14	
(Ca_0.17_Mn_0.83_)CO_3_^a^	2400	7.90	0.9976	0.0102	1.8802	− 10.01	− 10.02	− 868.28	− 868.33
(Ca_0.16_Mn_0.84_)CO_3_^b^	4800	7.93	1.0205	0.0092	1.9245	− 10.01	± 0.02	− 868.26	± 0.11
	7200	7.86	1.0598	0.0101	1.9521	− 10.04		− 868.44	
(Ca_0.08_Mn_0.92_)CO_3_^a^	2400	7.65	0.6258	0.0256	1.3296	− 10.21	− 10.23	− 840.16	− 840.23
(Ca_0.07_Mn_0.93_)CO_3_^b^	4800	7.66	0.7201	0.0199	1.5022	− 10.26	± 0.03	− 840.40	± 0.17
	7200	7.75	0.7722	0.0169	1.5735	− 10.21		− 840.13	
(Ca_0.00_Mn_1.00_)CO_3_	2400	7.29	0.0000	0.1871	0.5449	− 10.16	− 10.26	− 813.92	− 814.46
	4800	7.31	0.0000	0.1419	0.5413	− 10.26	± 0.10	− 814.47	± 0.54
	7200	7.34	0.0000	0.1380	0.4123	− 10.35		− 815.00	

^a^Bulk composition before dissolution; ^b^Bulk composition after 300 d of dissolution;

^c^IAP estimated for the bulk composition after 300 d of dissolution

**Table 3:** Analytical data and solubility of the (Ca_1-x_Mn_x_)CO_3_ solid solutions in N_2_-degassed water at 25 °C

Sample	Dissolution time (h)	pH	Concentration (mmol/L)			log_IAP^c^	Average log_IAP	ΔG_*f*_° (kJ/mol)	Average ΔG_*f*_° (kJ/mol)
			Ca	Mn	HCO_3_ + CO_3_				
(Ca_1.00_Mn_0.00_)CO_3_	2400	8.15	0.6712	0.0000	1.2626	− 8.40	− 8.46	− 1129.41	− 1129.71
	4800	8.14	0.6405	0.0000	1.1771	− 8.46	± 0.06	− 1129.73	± 0.30
	7200	8.12	0.6146	0.0000	1.1512	− 8.51		− 1129.99	
(Ca_0.89_Mn_0.11_)CO_3_^a^	2400	8.14	0.7306	0.0029	1.3955	− 8.63	− 8.70	− 1094.74	− 1095.12
(Ca_0.88_Mn_0.12_)CO_3_^b^	4800	8.11	0.6762	0.0021	1.2796	− 8.74	± 0.07	− 1095.36	± 0.38
	7200	8.13	0.6635	0.0019	1.2983	− 8.72		− 1095.27	
(Ca_0.78_Mn_0.22_)CO_3_^a^	2400	8.07	0.9646	0.0041	1.9198	− 8.74	− 8.83	− 1059.45	− 1059.96
(Ca_0.76_Mn_0.24_)CO_3_^b^	4800	8.05	0.8793	0.0028	1.7496	− 8.86	± 0.09	− 1060.14	± 0.51
	7200	8.02	0.8767	0.0027	1.7703	− 8.89		− 1060.30	
(Ca_0.68_Mn_0.32_)CO_3_^a^	2400	7.99	1.0529	0.0053	2.0362	− 8.98	− 9.03	− 1028.24	− 1028.56
(Ca_0.66_Mn_0.34_)CO_3_^b^	4800	8.00	0.9690	0.0041	1.9116	− 9.05	± 0.06	− 1028.66	± 0.32
	7200	8.00	0.9715	0.0037	1.8750	− 9.07		− 1028.78	
(Ca_0.58_Mn_0.42_)CO_3_^a^	2400	8.00	1.0290	0.0060	1.9608	− 9.21	− 9.26	− 996.92	− 997.17
(Ca_0.55_Mn_0.45_)CO_3_^b^	4800	7.97	1.0181	0.0053	1.9182	− 9.28	± 0.05	− 997.29	± 0.25
	7200	7.98	1.0265	0.0048	1.9362	− 9.28		− 997.30	
(Ca_0.47_Mn_0.53_)CO_3_^a^	2400	7.93	1.1697	0.0080	2.2236	− 9.37	− 9.44	− 962.15	− 962.54
(Ca_0.45_Mn_0.55_)CO_3_^b^	4800	7.92	1.1162	0.0066	2.0954	− 9.46	± 0.07	− 962.62	± 0.38
	7200	7.96	1.0650	0.0054	1.9879	− 9.49		− 962.83	
(Ca_0.37_Mn_0.63_)CO_3_^a^	2400	7.96	1.4048	0.0107	2.0743	− 9.49	− 9.61	− 930.26	− 930.93
(Ca_0.35_Mn_0.65_)CO_3_^b^	4800	7.89	1.2750	0.0078	2.0165	− 9.67	± 0.12	− 931.27	± 0.67
	7200	7.92	1.1846	0.0073	2.0147	− 9.66		− 931.26	
(Ca_0.28_Mn_0.72_)CO_3_^a^	2400	7.92	1.0190	0.0104	1.9639	− 9.75	− 9.82	− 902.58	− 902.97
(Ca_0.27_Mn_0.73_)CO_3_^b^	4800	7.90	1.0256	0.0081	1.9219	− 9.85	± 0.07	− 903.18	± 0.39
	7200	7.90	1.0883	0.0077	2.0129	− 9.85		− 903.15	
(Ca_0.17_Mn_0.83_)CO_3_^a^	2400	8.00	0.9781	0.0124	1.8736	− 9.84	− 9.98	− 867.32	− 868.09
(Ca_0.16_Mn_0.84_)CO_3_^b^	4800	7.91	0.9155	0.0102	1.7284	− 10.04	± 0.13	− 868.42	± 0.76
	7200	7.88	1.0614	0.0092	1.9629	− 10.05		− 868.51	
(Ca_0.08_Mn_0.92_)CO_3_^a^	2400	7.85	0.6178	0.0246	1.3181	− 10.01	− 10.11	− 839.09	− 839.66
(Ca_0.08_Mn_0.92_)CO_3_^b^	4800	7.88	0.6963	0.0145	1.4213	− 10.16	± 0.10	− 839.95	± 0.56
	7200	7.86	0.7745	0.0140	1.5604	− 10.16		− 839.93	
(Ca_0.00_Mn_1.00_)CO_3_	2400	7.31	0.0000	0.1700	0.5620	− 10.17	− 10.25	− 813.95	− 814.39
	4800	7.34	0.0000	0.1576	0.4657	− 10.25	± 0.08	− 814.39	± 0.44
	7200	7.29	0.0000	0.1489	0.4647	− 10.33		− 814.84	

^a^Bulk composition before dissolution; ^b^Bulk composition after 300 d of dissolution

^c^IAP estimated for the bulk composition after 300 d of dissolution

**Table 4:** Analytical data and solubility of the (Ca_1-x_Mn_x_)CO_3_ solid solutions in CO_2_-saturated water at 25 °C

Sample	Dissolution time (h)	pH	Concentration (mmol/L)			log_IAP^c^	Average log_IAP	ΔG_*f*_° (kJ/mol)	Average ΔG_*f*_° (kJ/mol)
			Ca	Mn	HCO_3_ + CO_3_				
(Ca_1.00_Mn_0.00_)CO_3_	2400	8.00	0.7096	0.0000	1.3379	− 8.51	− 8.59	− 1130.00	− 1130.48
	4800	7.92	0.6304	0.0000	1.1650	− 8.69	± 0.10	− 1131.05	± 0.57
	7200	8.04	0.6315	0.0000	1.1547	− 8.57		− 1130.38	
(Ca_0.89_Mn_0.11_)CO_3_^a^	2400	8.13	0.7705	0.0030	1.4653	− 8.60	− 8.66	− 1094.57	− 1094.93
(Ca_0.88_Mn_0.12_)CO_3_^b^	4800	8.14	0.6686	0.0032	1.2606	− 8.69	± 0.06	− 1095.12	± 0.36
	7200	8.16	0.6512	0.0033	1.2451	− 8.69		− 1095.09	
(Ca_0.78_Mn_0.22_)CO_3_^a^	2400	8.11	0.7954	0.0039	1.5584	− 8.85	− 8.92	− 1060.05	− 1060.50
(Ca_0.76_Mn_0.24_)CO_3_^b^	4800	8.11	0.6830	0.0039	1.3393	− 8.95	± 0.08	− 1060.66	± 0.45
	7200	8.12	0.6593	0.0038	1.2771	− 8.97		− 1060.79	
(Ca_0.68_Mn_0.32_)CO_3_^a^	2400	8.06	0.8304	0.0054	1.5895	− 9.08	− 9.05	− 1028.73	− 1028.53
(Ca_0.65_Mn_0.35_)CO_3_^b^	4800	8.09	0.8764	0.0046	1.6778	− 9.04	± 0.03	− 1028.49	± 0.20
	7200	8.05	0.9419	0.0048	1.8336	− 9.02		− 1028.38	
(Ca_0.58_Mn_0.42_)CO_3_^a^	2400	8.06	0.9222	0.0057	1.7134	− 9.24	− 9.23	− 997.07	− 997.03
(Ca_0.55_Mn_0.45_)CO_3_^b^	4800	8.06	0.9464	0.0054	1.7810	− 9.23	± 0.01	− 997.00	± 0.04
	7200	8.01	1.0500	0.0052	1.9438	− 9.23		− 997.02	
(Ca_0.47_Mn_0.53_)CO_3_^a^	2400	8.01	0.8893	0.0058	1.6451	− 9.55	− 9.47	− 963.04	− 962.60
(Ca_0.44_Mn_0.56_)CO_3_^b^	4800	8.01	1.0871	0.0069	2.0502	− 9.39	± 0.08	− 962.13	± 0.46
	7200	7.99	1.0713	0.0058	1.9531	− 9.48		− 962.61	
(Ca_0.37_Mn_0.63_)CO_3_^a^	2400	7.98	1.2446	0.0101	1.6866	− 9.58	− 9.61	− 930.78	− 930.96
(Ca_0.35_Mn_0.65_)CO_3_^b^	4800	7.95	1.3037	0.0085	1.8842	− 9.61	± 0.03	− 930.94	± 0.19
	7200	7.99	1.1368	0.0070	1.8409	− 9.64		− 931.14	
(Ca_0.28_Mn_0.72_)CO_3_^a^	2400	8.00	0.9182	0.0105	1.7070	− 9.77	− 9.82	− 902.46	− 902.74
(Ca_0.25_Mn_0.75_)CO_3_^b^	4800	8.02	0.8777	0.0084	1.6451	− 9.84	± 0.05	− 902.85	± 0.28
	7200	8.02	0.9792	0.0073	1.7481	− 9.85		− 902.92	
(Ca_0.17_Mn_0.83_)CO_3_^a^	2400	7.81	0.9402	0.0105	1.7566	− 10.12	− 10.05	− 868.91	− 868.51
(Ca_0.16_Mn_0.84_)CO_3_^b^	4800	7.97	0.8331	0.0108	1.5837	− 10.00	± 0.07	− 868.19	± 0.40
	7200	8.01	0.8028	0.0091	1.4962	− 10.04		− 868.44	
(Ca_0.08_Mn_0.92_)CO_3_^a^	2400	7.70	0.5699	0.0264	1.2909	− 10.16	− 10.24	− 839.86	− 840.32
(Ca_0.07_Mn_0.93_)CO_3_^b^	4800	7.71	0.6976	0.0196	1.4666	− 10.22	± 0.11	− 840.18	± 0.60
	7200	7.71	0.6368	0.0174	1.1982	− 10.35		− 840.93	
(Ca_0.00_Mn_1.00_)CO_3_	2400	7.25	0.0000	0.1717	0.5410	− 10.25	− 10.28	− 814.39	− 814.59
	4800	7.24	0.0000	0.1687	0.4840	− 10.31	± 0.03	− 814.76	± 0.20
	7200	7.29	0.0000	0.1633	0.4620	− 10.29		− 814.64	

^a^Bulk composition before dissolution; ^b^Bulk composition after 300 d of dissolution;

^c^IAP estimated for the bulk composition after 300 d of dissolution

Regarding the dissolution in N_2_-degassed, air-saturated and CO_2_-saturated ultrapure waters at 25 °C, the average log_IAP values at the final steady state (≈log_*K*_sp_) were estimated to be − 8.46 ± 0.06, − 8.44 ± 0.10 and -8.59 ± 0.10, respectively, for calcite [CaCO_3_] with Δ*G*_*f*_° values of − 1129.71 ± 0.30 kJ/mol, − 1129.63 ± 0.59 kJ/mol and − 1130.48 ± 0.57 kJ/mol, respectively. These results were consistent with the various *K*_sp_ and Δ*G*_*f*_° values for CaCO_3_ found in the literature. For instance, both the minteq.v4.dat database and the phreeqc.dat database showed a log_*K*_sp_ of − 8.48 [21, 36, 37]. Additionally, CaCO_3_ has a very well-defined solubility product (log_*K*_sp_) reported to fall within − 8.30 [14, 38].

Regarding the dissolution in N_2_-degassed, air-saturated and CO_2_-saturated ultrapure waters at 25 °C, the average log_IAP values at the final steady state (≈log_*K*_sp_) were estimated to be − 10.25 ± 0.08, − 10.26 ± 0.10 and − 10.28 ± 0.03, respectively, for rhodochrosite [MnCO_3_] with Δ*G*_*f*_° values of − 814.39 ± 0.44 kJ/mol, − 814.46 ± 0.54 kJ/mol and − 814.59 ± 0.20 kJ/mol, respectively. Many inconsistent values of the solubility product for rhodochrosite [MnCO_3_] have been reported in the literature [11]. Some of the log_*K*_sp_ values for MnCO_3_ reported are − 9.47 [5], − 10.06 [39], − 10.30 [3], − 10.41 [13], − 10.42 [40], − 10.55 [41], − 10.62 [42], − 10.76 [43], − 10.99 [14], − 11.39 ~ − 11.65 [38] and − 12.19 [44]. The various Δ*G*_*f*_° values for MnCO_3_(s) found in the literature ranged from − 809.89 ± 0.73 kJ/mol [5] to − 818.81 kJ/mol [14], including − 812.53 kJ/mol [45], − 813.0 kJ/mol [39], − 814.6 kJ/mol [14], − 816.05 ± 1.38 kJ/mol [46], − 816.32 ± 0.07 kJ/mol [41], − 816.6 ± 0.2 kJ/mol [47] and − 816.7 kJ/mol [42]. As a result, a large difference among the log_*K*_sp_ values for rhodochrosite could also be found in various databases. For instance, whereas the minteq.v4.dat database [36] showed a log_*K*_sp_ of − 10.58, the phreeqc.dat database had a log_*K*_sp_ of − 11.13 [21, 37]. A log_*K*_sp_ of − 10.50 is usually accepted in the geochemical literature [48]. The disparity in the published thermodynamic solubility products arose from the lack of experimental analyses and their derivation/extrapolation, from the differences in the involved aqueous speciations [49] or from the impurities in solid samples [5].

As the X_Mn_ of the (Ca_1-x_Mn_x_)CO_3_ solid solutions increased, the log_IAP values at the final steady state decreased from − 8.44 ~ − 8.59 for calcite to − 10.25 ~ − 10.28 for rhodochrosite (Tables 2, 3 and 4). These values were consistent with log_*K*_sp_ = − 9.3 for Mn_25_Ca_0.75_CO_3_ and log_IAP of − 9.9 for Mn_0.48_Ca_0.52_CO_3_ obtained from dissolution experiments [4]. The disordered calcian rhodochrosite (Mn_0.58_Ca_0.39_Mg_0.03_CO_3_) precipitated in the laboratory showed a log_IAP value of -10.38 after long-term equilibration [6]. The solubilities of the Mn_x_Ca_1-x_CO_3_ solid solutions in the present work were generally lower than the data calculated using the empirical expression derived from the experimental electrochemical cell data [5], which was related to the Δ*G*_*f*_° value of − 809.89 ± 0.73 kJ/mol for MnCO_3_ being too large in comparison to those (− 812.53 ~ − 818.81 kJ/mol) in the present work and other studies [14, 39, 41, 42, 45–47].

### Lippmann diagram

#### Construction of the Lippmann diagram

The solid solution–aqueous solution (SS-AS) system is of essential importance to understand the geochemical process. Despite many studies, thermodynamic data about the SS-AS interaction are still scarce. The methods to construct Lippmann diagrams have been described in detail in numerous works covering a wide variety of SS-AS systems [2, 10, 25, 26, 28, 34, 50–56].

The Lippmann diagram is a graph that shows the “*solidus*” and “*solutus*” phase relationship in an SS-AS system. The “total activity product” (ΣΠ_SS_) is defined as the sum of the partial activity products of the endmembers at equilibrium. The “*solidus*” and “*solutus*" curves are the plots of ΣΠ_SS_ versus the solid component and the solution component, which define all probable thermodynamic saturation states as a function of the solid and aqueous components, respectively.

Regarding the calcite-rhodochrosite solid solutions [(Ca_1-x_Mn_x_)CO_3_], the “*solidus*” curve is described by

11 $$\sum {\prod_{{{\text{SS}}}} } = \left( {\left\{ {{\text{Ca}}^{{{2} + }} } \right\} + \left\{ {{\text{Mn}}^{{{2} + }} } \right\}} \right)\left\{ {{\text{CO}}_{{3}}^{{{2} - }} } \right\} \, = {\text{K}}_{{{\text{Ca}}}} {\text{X}}_{{{\text{Ca}}}} \gamma_{{{\text{Ca}}}} + {\text{K}}_{{{\text{Mn}}}} {\text{X}}_{{{\text{Mn}}}} \gamma_{{{\text{Mn}}}}$$

where {} designates aqueous activity. ${\text{K}}_{\text{Ca}}$ and ${\text{K}}_{\text{Mn}}$, ${\text{X}}_{\text{Ca}}$ and ${\text{X}}_{\text{Mn}}$, ${\gamma }_{\text{Ca}}$ and ${\gamma }_{\text{Mn}}$ are the solubility products, the molar ratios (x, 1−x) and the activity coefficients of CaCO_3_ and MnCO_3_ in the (Ca_1-x_Mn_x_)CO_3_ solid solutions, respectively.

The “*solutus*” curve is expressed by:

12 $$\sum {\prod_{{{\text{SS}}}} } = \frac{1}{{\frac{{{\text{X}}_{{{\text{Ca}}^{{2 + }} {\text{,aq}}}} }}{{{\text{K}}_{{{\text{Ca}}}} \gamma_{{{\text{Ca}}}} }} + \frac{{{\text{X}}_{{{\text{Mn}}^{{2 + }} {\text{,aq}}}} }}{{{\text{K}}_{{{\text{Mn}}}} \gamma_{{{\text{Mn}}}} }}}}$$

where ${\text{X}}_{{\text{Ca}}^{2+}\text{,aq}}$ and ${\text{X}}_{{\text{Mn}}^{2+}\text{,aq}}$ are the activity ratios of aqueous Ca^2+^ and Mn^2+^, respectively.

Regarding the members of fixed X_Ca_ = 1-X_Mn_, a set of minimum stoichiometric saturation curves in terms of ${\text{X}}_{{\text{Ca}}^{2+}\text{,aq}}$ are expressed by:

13 $$\sum {\prod_{{{\text{SS}}}} } = \frac{{{\text{IAP}}}}{{({\text{X}}_{{{\text{Ca}}^{{2 + }} {\text{,aq}}}} )^{{{\text{X}}_{{{\text{Ca}}}} }} ({\text{X}}_{{{\text{Mn}}^{{2 + }} {\text{,aq}}}} )^{{{\text{X}}_{{{\text{Mn}}}} }} }}$$

Regarding the endmembers CaCO_3_ and MnCO_3_, X_Ca_ = 1 and X_Ca_ = 0, the endmember saturation curves are described by:

14 $$\sum {\prod_{{{\text{CaCO}}_{{3}} }} } = \frac{{\left\{ {{\text{Ca}}^{{2 + }} } \right\}\left\{ {{\text{CO}}_{{3}}^{{2 - }} } \right\}}}{{({\text{X}}_{{{\text{Ca}}^{{2 + }} {\text{,aq}}}} )^{{{\text{X}}_{{{\text{Ca}}}} }} }} = \frac{{{\text{K}}_{{{\text{Ca}}}} }}{{({\text{X}}_{{{\text{Ca}}^{{2 + }} {\text{,aq}}}} )^{{{\text{X}}_{{{\text{Ca}}}} }} }}$$

15 $$\sum {\prod_{{{\text{MnCO}}_{{3}} }} = \frac{{\left\{ {{\text{Mn}}^{{2 + }} } \right\}\left\{ {{\text{CO}}_{3}^{2 - } } \right\}}}{{\left( {{\text{X}}_{{{\text{Mn}}^{2 + } ,{\text{aq}}}} } \right)^{{{\text{X}}_{{{\text{Mn}}}} }} }} = \frac{{{\text{K}}_{{{\text{Mn}}}} }}{{\left( {{\text{X}}_{{{\text{Mn}}^{{2 + }} ,{\text{aq}}}} } \right)^{{{\text{X}}_{{{\text{Mn}}}} }} }}}$$

### Lippmann diagram for the nonideal (Ca,Mn)CO_3_ solid solutions

Regarding the (Ca,Mn)CO_3_-H_2_O SS-AS system, the extent of nonideality and the stability field have been unequivocally established [2]. Several works reported componential ranges from the total miscibility between CaCO_3_ and MnCO_3_ to restricted solid solubilities with diverse ranges of miscibility gaps [57, 58]. CaCO_3_ and MnCO_3_ are suggested to form a complete series, but many of the intermediate Mn_x_Ca_1-x_CO_3_ compositions are metastable or unstable under ambient conditions [4]. Therefore, the (Ca,Mn)CO_3_ solid solution is accepted to be nonideal and incomplete at 25 °C; however, it is frequently considered regular. Thus, the estimated values of a_0_ found in the literature for this solid solution differ enormously. For example, a theoretically derived *a*_*0*_ =  +3.23 (25 °C) was proposed [10], while *a*_*0*_ = − 1 ± 3 at 20 °C was calculated from experimental solubility data [4]. A negative value of *a*_*0*_ (− 3.5 at 5 °C) was also estimated from observations on natural phase relations in marine anoxic sediments [18]. The *a*_0_ parameter was calculated from the miscibility gap [15] to be *a*_*0*_ < 2 at 25 °C [16]. Experimental studies under ambient conditions [5] and high temperatures [59] have shown that a regular model was probably insufficient to strictly describe thermodynamic equilibrium in this system. The two-parameter Guggenheim expansion equations (“subregular” models) were effectively applied to fit the experimental solubilities of (Sr,Ca)CO_3_-H_2_O, (Ba,Sr)CO_3_-H_2_O, and (Ca,Mg)CO_3_-H_2_O systems [16]. Therefore, in the absence of more precise data, the (Ca_1-x_Mn_x_)CO_3_ solid solutions were assumed to be subregular, and the two Guggenheim coefficients *a*_*0*_ and *a*_1_ were calculated in this work.

The solid activity coefficients of CaCO_3_ (${\gamma }_{\text{Ca}}$) and MnCO_3_ (${\gamma }_{\text{Mn}}$) in terms of solid components (1−x, x) can be estimated by the Redlich and Kister equations [50, 54, 60], which are expressed as:

16 $${\text{ln}} \gamma_{{{\text{Ca}}}} = \, \left( {{1} - {\text{x}}} \right)^{{2}} \left[ {a_{0} {-}a_{1} \left( {{\text{3x}} - \left( {{1} - {\text{x}}} \right)} \right) \, + \, \ldots } \right]$$

17 $$\ln \gamma_{{{\text{Mn}}}} = {\text{x}}^{{2}} \left[ {a_{0} {-}a_{1} \left( {{3}\left( {{1} - {\text{x}}} \right) - {\text{x}}} \right) \, + \ldots } \right]$$

where 1−x and x are the molar ratios (${\mathrm{X}}_{\text{Ca}}$ and ${\mathrm{X}}_{\text{Mn}}$) of CaCO_3_ and MnCO_3_ in the (Ca_1-x_Mn_x_)CO_3_ solid solutions, respectively.

When the stoichiometric saturation state was reached during dissolution, the excess free energy of mixing for the (B_x_C_1-x_)A solid solutions (*G*^E^) can be estimated with the following:

18 $$G^{{\text{E}}} = {\text{ RT}}\left[ {{\text{lnK}}_{{{\text{SS}}}} - {\text{ x }}\left( {{\text{lnK}}_{{{\text{CA}}}} + {\text{ lnx}}} \right) \, - \, \left( {{1} - {\text{x}}} \right) \, \left( {{\text{lnK}}_{{{\text{BA}}}} + {\text{ ln}}\left( {{1} - {\text{x}}} \right)} \right)} \right]$$

where K_SS_ is the stoichiometric saturation constant and K_CA_ and K_BA_ are the solubility products of CA and BA, respectively [34, 50]. The Guggenheim expansion equation for *G*^E^ is:

19 $$G^{{\text{E}}} = {\text{ x}}\left( {{1} - {\text{x}}} \right){\text{ RT }}\left[ {a_{0} + a_{1} \left( {{\text{x}} - \left( {{1} - {\text{x}}} \right)} \right) \, + a_{2} \left( {{\text{x}} - \left( {{1} - {\text{x}}} \right)} \right)^{{2}} \ldots } \right]$$

where *a*_*0*_*, a*_*1*_ and *a*_*2*_ are dimensionless Guggenheim coefficients that usually express the excess free energy of solid solutions in terms of their solid components. Combining Eqs. (18) and (19) and rewriting them for the (Ca_1-x_Mn_x_)CO_3_ solid solutions while using only the coefficients *a*_*0*_ and *a*_1_ yields:

20 $${\text{Ln}}K_{{{\text{sp}}}} = {\text{ x}}\left( {{1} - {\text{x}}} \right)a_{0} + {\text{ x}}\left( {{1} - {\text{x}}} \right)\left( {{\text{x}} - \left( {{1} - {\text{x}}} \right)} \right)a_{1} + \, \left( {{1} - {\text{x}}} \right){\text{ ln}}\left[ {\left( {{1} - {\text{x}}} \right){\text{ K}}_{{{\text{Ca}}}} } \right] \, + {\text{ x ln}}\left[ {{\text{x K}}_{{{\text{Mn}}}} } \right)]$$

When the stoichiometric saturation state was reached during dissolution, *a*_*0*_ and *a*_1_ can be calculated by:

21 $${\text{Ln}}K_{{{\text{sp}}}} = {\text{ x}}\left( {{1} - {\text{x}}} \right)a_{0} + {\text{ x}}\left( {{1} - {\text{x}}} \right)\left( {{\text{x}} - \left( {{1} - {\text{x}}} \right)} \right)a_{1} + \, \left( {{1} - {\text{x}}} \right){\text{ ln}}\left[ {\left( {{1} - {\text{x}}} \right){\text{ K}}_{{{\text{Ca}}}} } \right] \, + {\text{ x ln}}\left[ {{\text{x K}}_{{{\text{Mn}}}} } \right)]$$

The log_IAP values (≈log_*K*_sp_) for the final three solutions after 2400–7200 h dissolution in N_2_-degassed, air-saturated and CO_2_-saturated ultrapure waters were plotted versus X_Mn_ of the (Ca_1-x_Mn_x_)CO_3_ solid solutions in Additional file 1: Appendix S9, showing that the experimental log_*K*_sp_ values were near and slightly lower than those of an ideal (Ca_1-x_Mn_x_)CO_3_ solid solution. The *K*_sp_ values as a function of X_Mn_ could be very well fitted to Eq. (21) with the two-parameter Guggenheim models of *a*_*0*_ = 2.96 and *a*_*1*_ = − 0.20, *a*_*0*_ = 2.92 and *a*_*1*_ = − 0.33 and *a*_*0*_ = 3.28 and *a*_*1*_ = 0.61, respectively. Moreover, the dimensionless Guggenheim parameters were also estimated based on the miscibility gap mole fractions of MnCO_3_ (X_Mn_ = 0.11 and 0.83) at 25 °C after the powder XRD measurements by using the computer code MBSSAS [61] to be *a*_*0*_ = 2.52 and *a*_*1*_ = − 0.19. It is obvious that the estimated Guggenheim parameters in the present work were comparable to *a*_*0*_ =  + 3.23 [10] and were therefore chosen to construct the Lippmann diagrams of the (Ca_1-x_Mn_x_)CO_3_ solid solutions.

### Solid solution–aqueous solution interaction

**Fig. 9:**
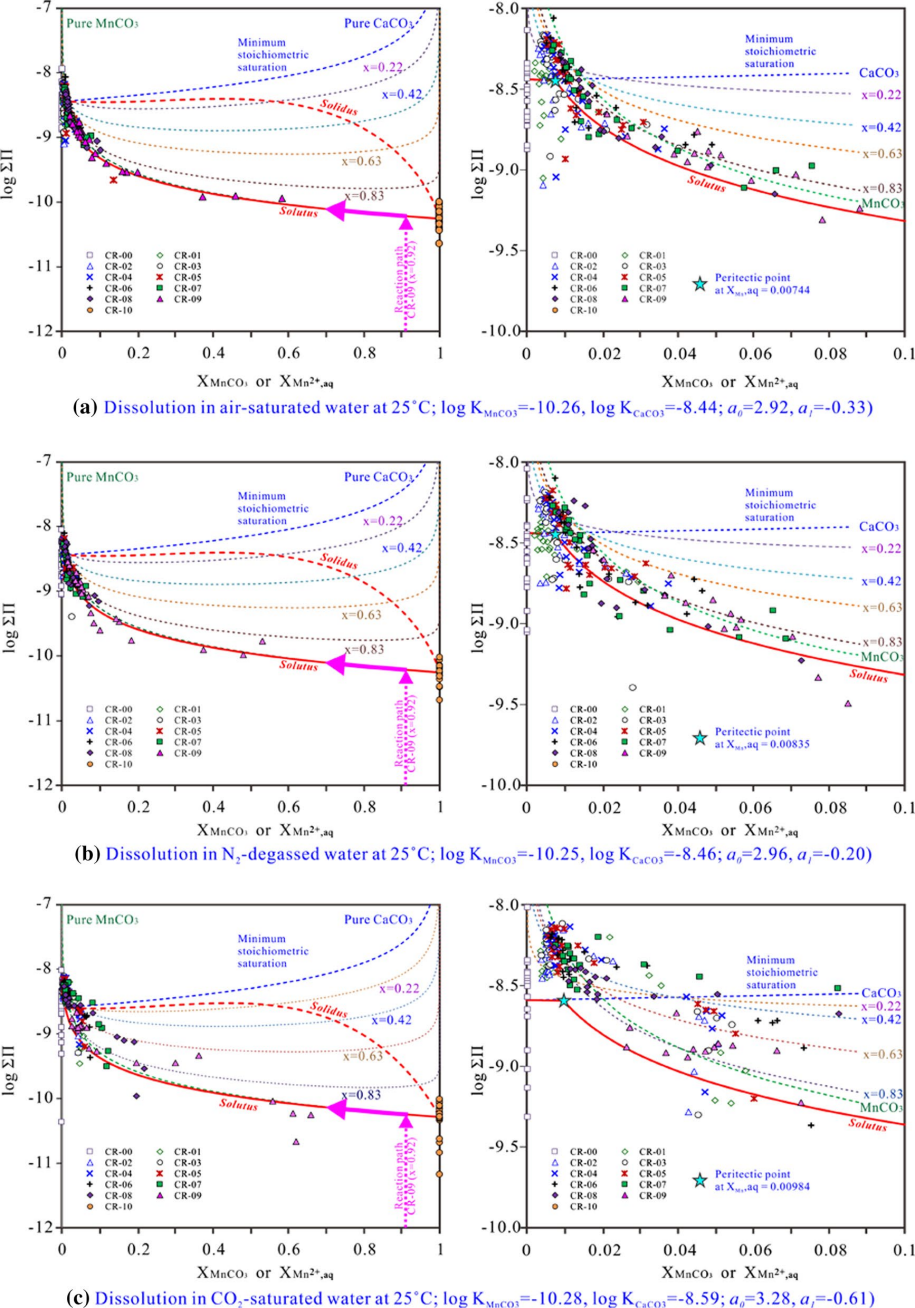
Lippmann diagram of the nonideal (Ca_1-x_Mn_x_)CO_3_ solid solutions together with the plot of some stoichiometric saturation curves and the experimental data

The study of the precipitation behavior in the (Ca,Mn)CO_3_-H_2_O system at 25 °C was stated in the literature as being quite problematic [2, 11]. The main reason was that several authors report different values for the solubility product of rhodochrosite (log_K_MnCO3_) that vary between − 9.47 and − 12.19 [5, 44]. As a result, there existed a significant discrepancy concerning the calculation of the *solidus* and *solutus* curves [2]. Figure 9a illustrates the Lippmann diagrams of the (Ca_1-x_Mn_x_)CO_3_ solid solutions at 25 °C, computed with the subregular solid solution (*a*_*0*_ = 2.97 and *a*_*1*_ = − 0.27) and the calcite and rhodochrosite endmembers from the present study (log_K_CaCO3_ = − 8.44 and log_K_MnCO3_ = − 10.26, respectively). Along with the *solutus* and *solidus* curves, the stoichiometric saturation curves of pure CaCO_3_ (X_Mn_ = 0), the (Ca_1-x_Mn_x_)CO_3_ solid solutions (X_Mn_ = 0.21, 0.42, 0.61 and 0.82) and pure MnCO_3_ (X_Mn_ = 1) were also plotted in the Lippmann diagram, which was characterized by the peritectic point at ${\text{X}}_{{\text{Mn}}^{2+}\text{,aq}}$ = 0.00744. At this point, the aqueous component was in equilibrium with two solid phases (X_Mn_ = 0.04 and 0.57) at the same time, which defined the two extremes of the miscibility gap [2].

Additionally, the data of this work were also plotted as ({Ca^2+^} + {Mn^2+^}){CO_3_^2−^} versus ${\text{X}}_{{\text{Mn}}^{2+}\text{,aq}}$ (Fig. 9). An increase in the released Ca^2+^ concentration and the highest Mn^2+^ concentration for the solids were generally viewed in the dissolution progress [4]. The experimental data points indicated that the (Ca_1-x_Mn_x_)CO_3_ solid solutions dissolved incongruently and moved progressively up to the *solutus* curve and then along the *solutus* curve and/or the saturation curve of pure MnCO_3_ from right to left. The aqueous Mn-poor solutions were finally in equilibrium with the MnCO_3_-rich solids. Because the dissolution of the (Ca_1-x_Mn_x_)CO_3_ solid solutions was superimposed with the formation of new solid phases with a larger X_Mn_ than the initial solids, the overall dissolution became progressively incongruent for the solid samples (CR-01–CR-09), as reported for Ca_0.75_Mn_0.25_CO_3_ and Ca_0.25_Mn_0.75_CO_3_ [4]. The congruent dissolution part for the solids (CR-01–CR-09) was not detected, most likely due to the very high effective surface of the initial solids that caused quick dissolution and acted as the seeds for forming new solid phases [4]. Generally, the saturation curve for MnCO_3_ and the *solutus* curve for the (Ca_1-x_Mn_x_)CO_3_ solid solutions were progressively approached and exceeded during the SS-AS interaction (Fig. 9). The saturation indexes (SI) for rhodochrosite [MnCO_3_] increased as X_Mn_ increased, and the aqueous solutions were saturated or oversaturated with rhodochrosite [MnCO_3_] at the end of dissolution at X_Mn_ > 0.5 (CR-05–CR-10). The aqueous solutions were finally slightly oversaturated with calcite [CaCO_3_], with the exception of CR-09 (X_Mn_ = 0.92). The highest SI value with respect to calcite [CaCO_3_] was observed at X_Mn_ = 0.63 (CR-06) and then decreased as X_Mn_ decreased or increased (Additional file 1: Appendix S10). These results provide deeper insight into the mechanisms of the geochemical cycle of manganese in the environment.

## Conclusions

The crystal morphologies of synthesized (Ca_1-x_Mn_x_)CO_3_ solid solutions varied from blocky spherical crystal aggregates to smaller spheres following an increasing incorporation of Mn in the solids. The crystals grown from starting aqueous solutions with almost equal concentrations of Mn^2+^ and Ca^2+^ exhibited double peaks that corresponded to the reflections of calcite and rhodochrosite peaks in the XRD patterns and showed clear oscillatory concentric zoning, indicating core-to-rim compositional and crystalline heterogeneities in the solids. The central part of the crystal was always relatively rich in Mn. Surrounding this core were successive Ca-rich and Mn-rich rings.

Regarding the dissolution of the (Ca_1-x_Mn_x_)CO_3_ solid solutions in N_2_-degassed ultrapure water and air-saturated water, the aqueous Ca and HCO_3_ + CO_3_ concentrations increased up to their highest values after 1240–2400 h. The aqueous Mn concentrations and the aqueous Mn/(Ca + Mn) mole ratios increased up to the highest value after 6–12 h of dissolution and then decreased gradually to a steady state. Regarding dissolution in CO_2_-saturated water, the aqueous Ca and Mn concentrations reached maximum values within 1 h and then decreased to the steady state. The aqueous pH and Mn concentrations increased with the increasing X_Mn_ of the (Ca_1-x_Mn_x_)CO_3_ solid solutions, while the aqueous Ca and HCO_3_ + CO_3_ concentrations showed the highest values at X_Mn_ = 0.53–0.63.

The average log_IAP values at the final steady state of dissolution (≈log_*K*_sp_) and the Δ*G*_*f*_° values were estimated to be − 8.44 ~ − 8.59 and − 1129.63 ~ − 1130.48 kJ/mol, respectively, for calcite [CaCO_3_] and − 10.25 ~ − 10.28 and − 814.39 ~ − 814.59 kJ/mol for rhodochrosite [MnCO_3_], respectively. As X_Mn_ increased, the log_IAP values almost linearly decreased.

The plotting of the experimental data as Lippmann diagrams for the subregular (Ca_1-x_Mn_x_)CO_3_ solid solutions indicated that the solids dissolved incongruently and moved progressively up to the *solutus* curve and then along the *solutus* curve and/or the saturation curve of pure MnCO_3_ from right to left. The microcrystalline cores of the spherical crystal aggregates were preferentially dissolved to form cavities while simultaneously precipitating Mn-rich hexagonal prisms.

## Supplementary Information

**Additional file 1: Appendix S1.** Major speciation reactions involved in the PHREEQC calculation. **Appendix S2-A.** Position variation of the strongest peak (104) with X_Mn_ of the (Ca_1-x_Mn_x_)CO_3_ solid solutions. **Appendix S2-B.** Diffraction patterns of the (Ca_1-x_Mn_x_)CO_3_ solid solutions after dissolution (a) in N_2_-degassed water and (b) in CO_2_-saturated water for 300 d. **Appendix S3-A.** SEM images of the (Ca_1-x_Mn_x_)CO_3_ solid solutions after dissolution in air-saturated water for 300 d. **Appendix S3-B.** SEM images of the (Ca_1-x_Mn_x_)CO_3_ solid solutions after dissolution in N_2_-degassed water for 300 d. **Appendix S3-C.** SEM images of the (Ca_1-x_Mn_x_)CO_3_ solid solutions after dissolution in CO_2_-saturated water for 300 d. **Appendix S4.** BSE images of the equatorial sections and the corresponding compositional profiles along the A-B line of the (a) (Ca_0.68_Mn_0.32_)CO_3_ (CR-03) and (b) (Ca_0.48_Mn_0.52_)CO_3_ (CR-05) solids after dissolution in air-saturated water for 300 d. **Appendix S5.** BSE images of the equatorial sections and EDS analyses of the (Ca_0.68_Mn_0.32_)CO_3_ (CR-03) solid before and after dissolution in air-saturated water for 300 d to show that the microcrystalline sphere cores were preferentially dissolved to form hollows and Mn-rich hexagonal prisms. **Appendix S6.** Variation in the aqueous Mn/(Ca + Mn) mole ratios during the dissolution of the (Ca_1-x_Mn_x_)CO_3_ solid solutions. **Appendix S7.** XPS patterns of the (Ca_1-x_Mn_x_)CO_3_ solid solutions (a) before and (b) after dissolution in air-saturated water for 300 d. **Appendix S8.** Dependence of the aqueous components at the experimental end (300 d) on the X_Mn_ of (Ca_1-x_Mn_x_)CO_3_. **Appendix** **S9.** Estimation of the Guggenheim coefficients for the nonideal (Ca_1-x_Mn_x_)CO_3_ solid solutions. **Appendix S10.** Saturation indexes for calcite and rhodochrosite during the dissolution of the (Ca_1-x_Mn_x_)CO_3_ solid solutions.

## Data Availability

The datasets used for this manuscript are displayed in the figures in the manuscript and the additional file. The data in tabulated form are available upon request.

## Abbreviations

ICP-OES: Inductively Coupled Plasma-Optical Emission Spectrometry
XRD: X-ray Diffraction
ICDD: International Center for Diffraction Data
FE-SEM: Field Emission Scanning Electron Microscopy
BSEI: Backscattered Electron Imaging
PET: Polyethylene Terephthalate
IAP: Ion Activity Product
SS-AS: Solid Solution–Aqueous Solution
