# Supplementary material for: Dissolution and solubility of calcite-rhodochrosite solid solutions [(Ca1-xMnx)CO3] at 25 °C
Source: Geochem Trans. 2021 May 26;22:1. doi: 10.1186/s12932-021-00075-1 (PMC8157444; doi:10.1186/s12932-021-00075-1)
Supplement: Supplementary file 1 — Additional file 1: Appendix S1. Major speciation reactions involved in the PHREEQC calculation. Appendix S2-A. Position variation of the strongest peak (104) with XMn of the (Ca1-xMnx)CO3 solid solutions. Appendix S2-B. Diffraction patterns of the (Ca1-xMnx)CO3 solid solutions after dissolution (a) in N2-degassed water and (b) in CO2-saturated water for 300 d. Appendix S3-A. SEM images of the (Ca1-xMnx)CO3 solid solutions after dissolution in air-saturated water for 300 d. Appendix S3-B. SEM images of the (Ca1-xMnx)CO3 solid solutions after dissolution in N2-degassed water for 300 d. Appendix S3-C. SEM images of the (Ca1-xMnx)CO3 solid solutions after dissolution in CO2-saturated water for 300 d. Appendix S4. BSE images of the equatorial sections and the corresponding compositional profiles along the A-B line of the (a) (Ca0.68Mn0.32)CO3 (CR-03) and (b) (Ca0.48Mn0.52)CO3 (CR-05) solids after dissolution in air-saturated water for 300 d. Appendix S5. BSE images of the equatorial sections and EDS analyses of the (Ca0.68Mn0.32)CO3 (CR-03) solid before and after dissolution in air-saturated water for 300 d to show that the microcrystalline sphere cores were preferentially dissolved to form hollows and Mn-rich hexagonal prisms. Appendix S6. Variation in the aqueous Mn/(Ca + Mn) mole ratios during the dissolution of the (Ca1-xMnx)CO3 solid solutions. Appendix S7. XPS patterns of the (Ca1-xMnx)CO3 solid solutions (a) before and (b) after dissolution in air-saturated water for 300 d. Appendix S8. Dependence of the aqueous components at the experimental end (300 d) on the XMn of (Ca1-xMnx)CO3. Appendix S9. Estimation of the Guggenheim coefficients for the nonideal (Ca1-xMnx)CO3 solid solutions. Appendix S10. Saturation indexes for calcite and rhodochrosite during the dissolution of the (Ca1-xMnx)CO3 solid solutions. [file 12932_2021_75_MOESM1_ESM.pdf]

Appendix S1

Major speciation reactions involved in the PHREEQC calculation.

Speciation reactions	log_*K*
$Mn^{2+} + H_2O = MnOH^+ + H^+$	-10.597
$Mn^{2+} + 2H_2O = Mn(OH)_2^0 + 2H^+$	-22.2
$Mn^{2+} + 3H_2O = Mn(OH)_3^- + 3H^+$	-34.8
$Mn^{2+} + 4H_2O = Mn(OH)_4^{2-} + 4H^+$	-48.288
$Mn^{2+} + H^+ + CO_3^{2-} = MnHCO_3^+$	11.629
$Ca^{2+} + H_2O = CaOH^+ + H^+$	-12.697
$Ca^{2+} + H^+ + CO_3^{2-} = CaHCO_3^+$	11.599
$CO_3^{2-} + Ca^{2+} = CaCO_3^0$	3.2
$H^+ + CO_3^{2-} = HCO_3^-$	10.329
$2H^+ + CO_3^{2-} = H_2CO_3^0$	16.681
$MnCO_3$ (Rhodochrosite) $= Mn^{2+} + CO_3^{2-}$	-10.58
$Mn(OH)_2$ (Pyrochroite) $+ 2H^+ = Mn^{2+} + 2H_2O$	15.194
CaO (Lime) $+ 2H^+ = Ca^{2+} + H_2O$	32.6993
$Ca(OH)_2$ (Portlandite) $+ 2H^+ = Ca^{2+} + 2H_2O$	22.804
$CaCO_3$ (Calcite) $= Ca^{2+} + CO_3^{2-}$	-8.48
$CaCO_3$ (Aragonite) $= Ca^{2+} + CO_3^{2-}$	-8.3

Note: Compiled from the minteq.v4.dat database (Parkhurst and Appelo, 2013).

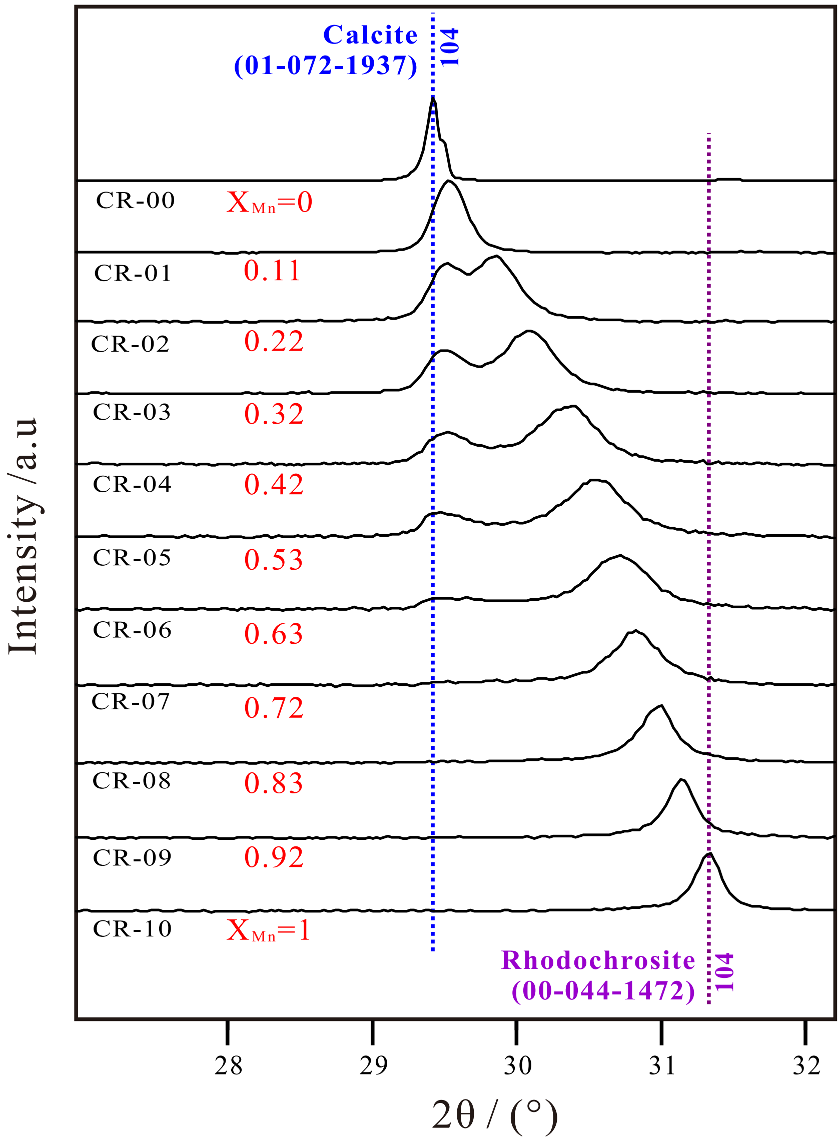

Appendix S2-A

Position variation of the strongest peak (104) with X_{Mn} of the $(Ca_{1-x}Mn_x)CO_3$ solid solutions.

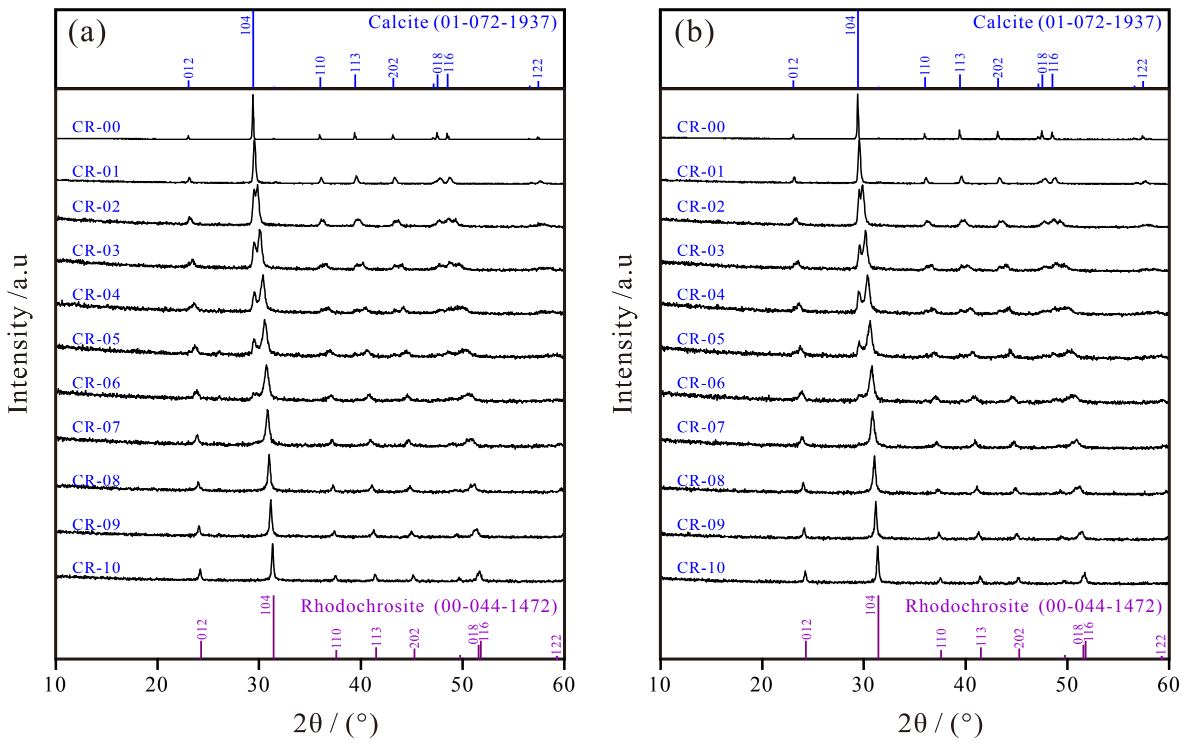

Appendix S2-B

Diffraction patterns of the $(Ca_{1-x}Mn_x)CO_3$ solid solutions after dissolution (a) in the N_2-degassed water and (b) in the CO_2-saturated water for 300d.

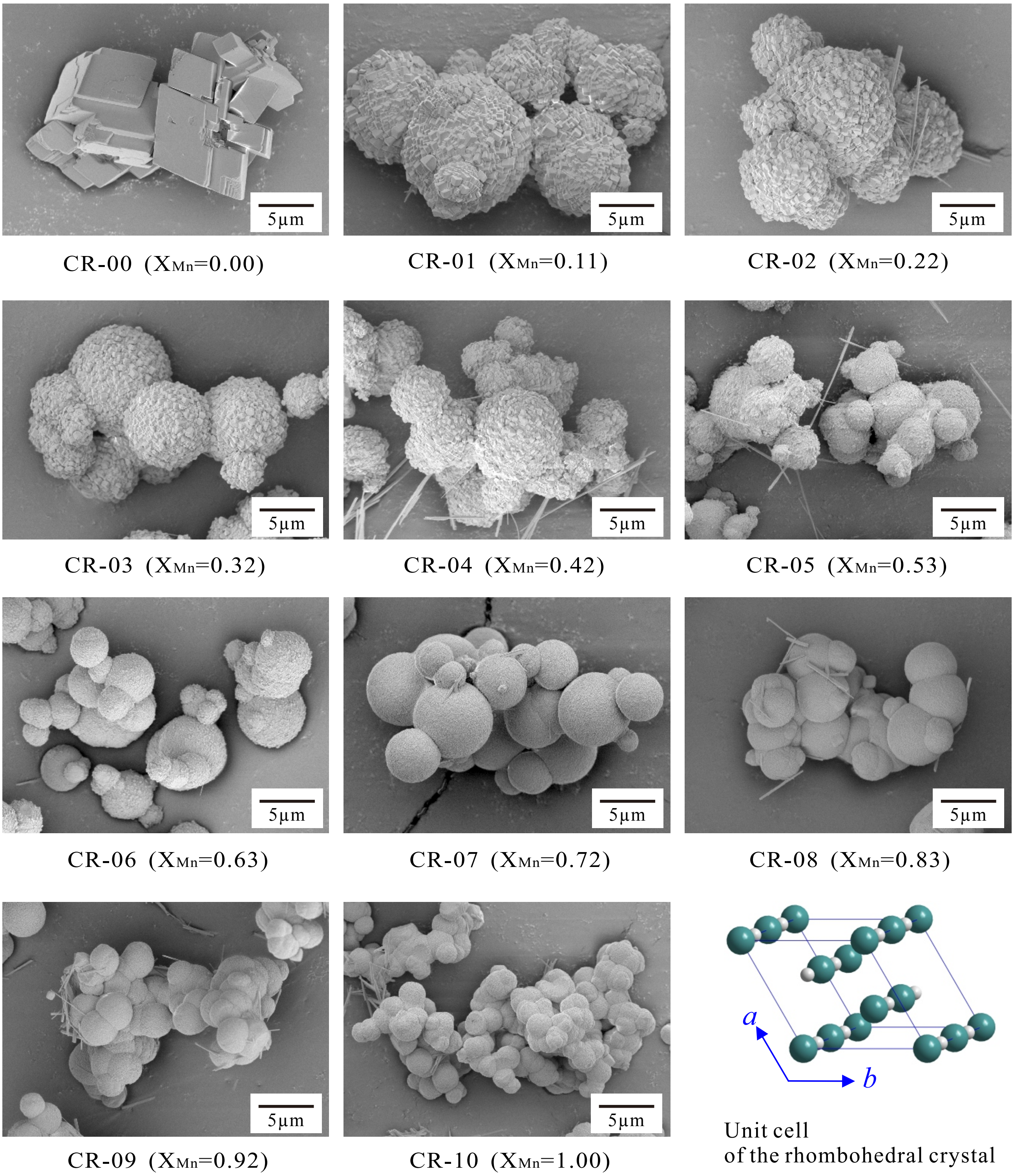

Appendix S3-A

SEM images of the $(Ca_{1-x}Mn_x)CO_3$ solid solutions after dissolution in the air-saturated water for 300d.

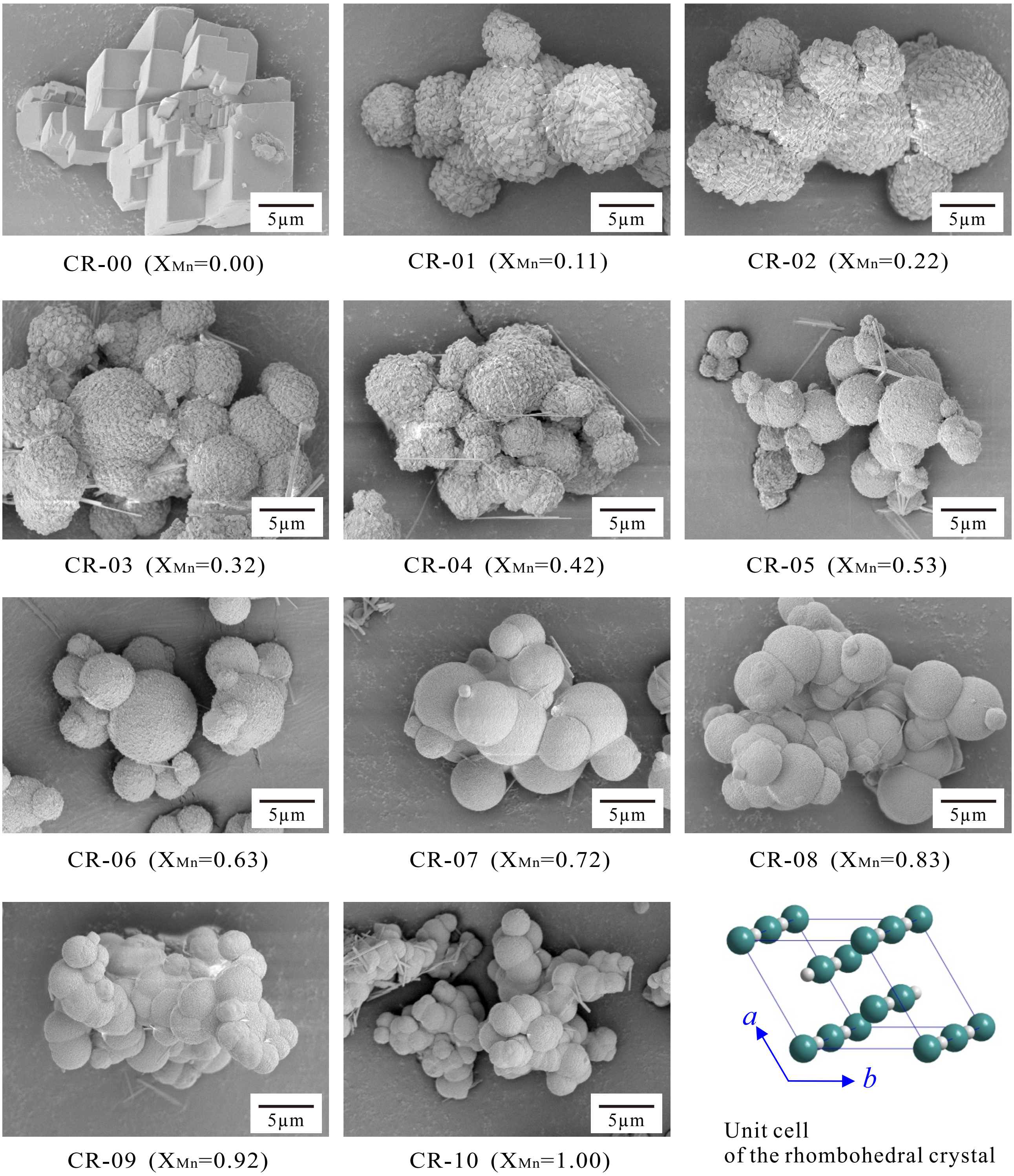

Appendix S3-B

SEM images of the $(Ca_{1-x}Mn_x)CO_3$ solid solutions after dissolution in the N_2-degassed water for 300d.

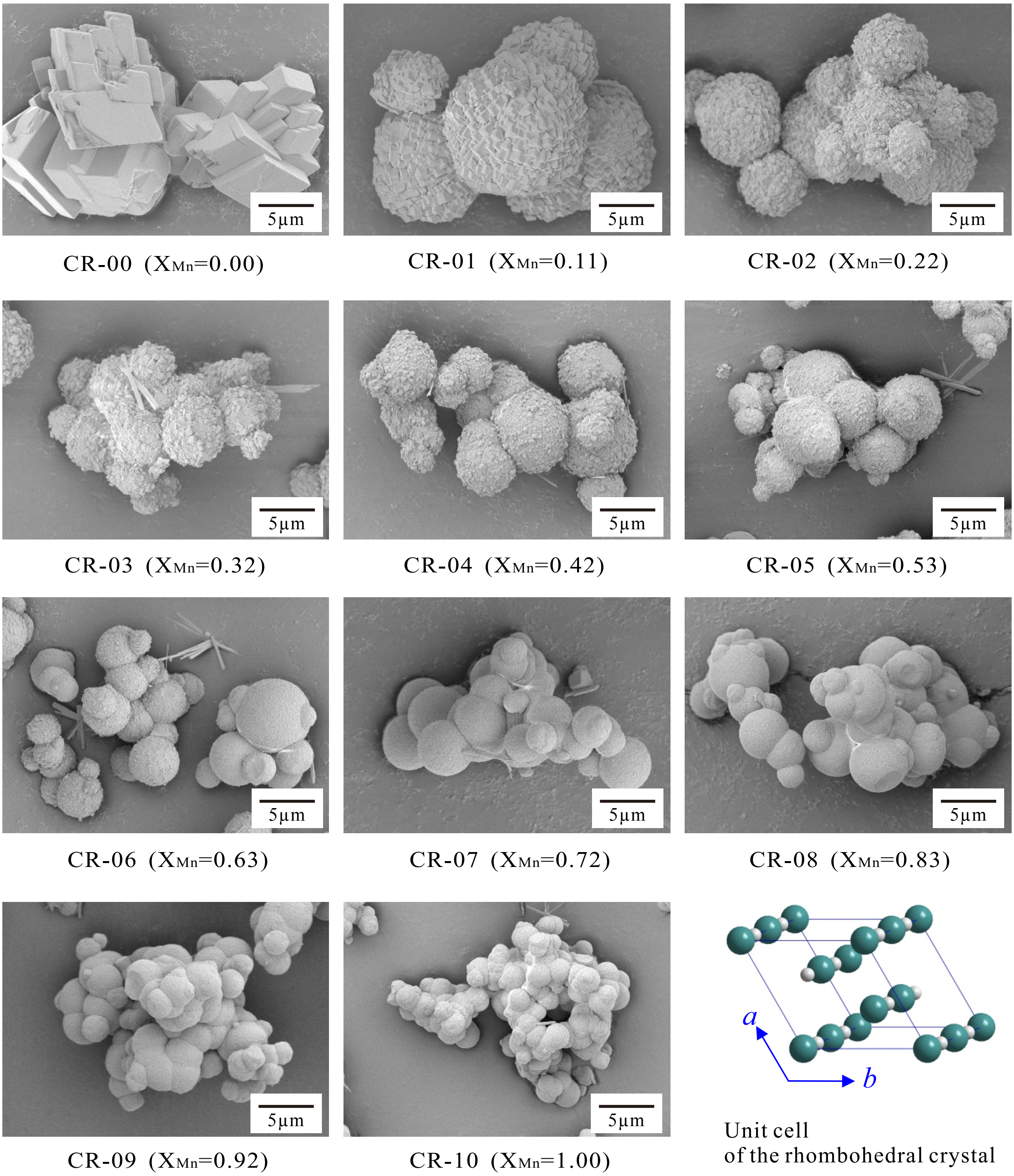

Appendix S3-C

SEM images of the $(Ca_{1-x}Mn_x)CO_3$ solid solutions after dissolution in the CO_2-saturated water for 300d.

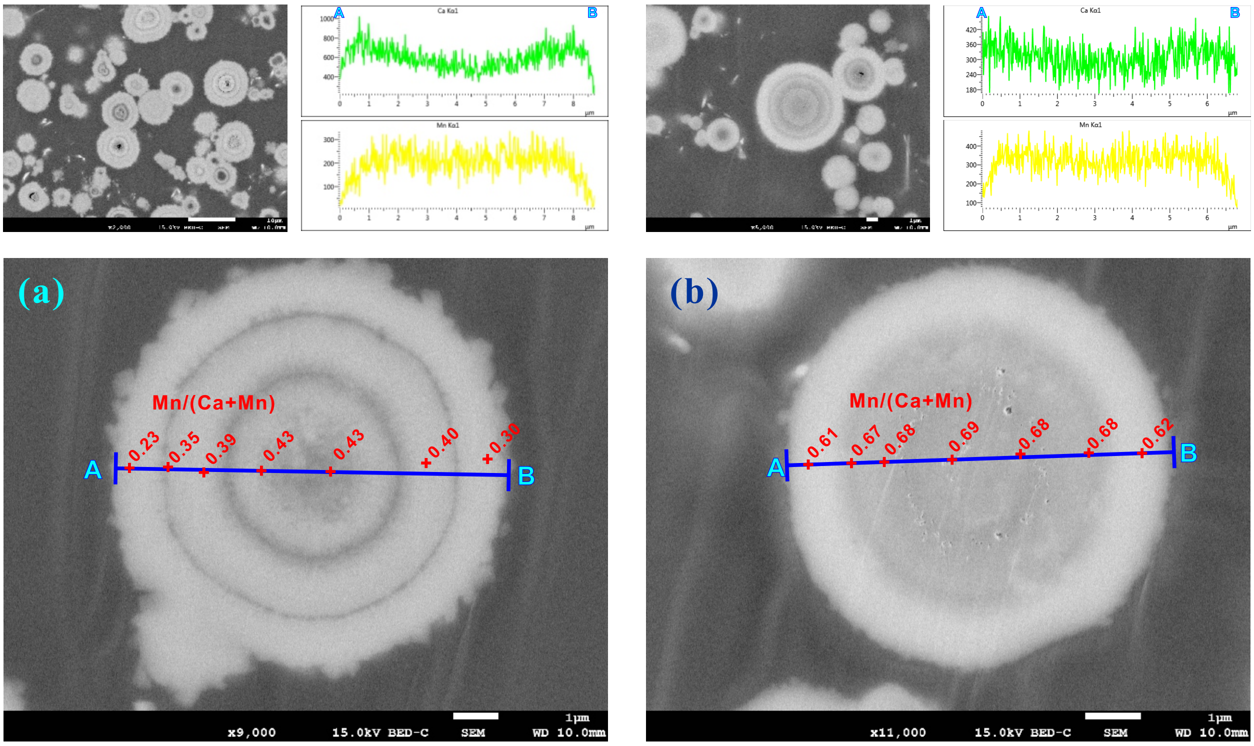

Appendix S4

BSE images of the equatorial sections and the corresponding compositional profiles along the A-B line of the solids (a) $(Ca_{0.68}Mn_{0.32})CO_3$ (CR-03) and (b) $(Ca_{0.48}Mn_{0.52})CO_3$ (CR-05) after dissolution in the air-saturated water for 300d.

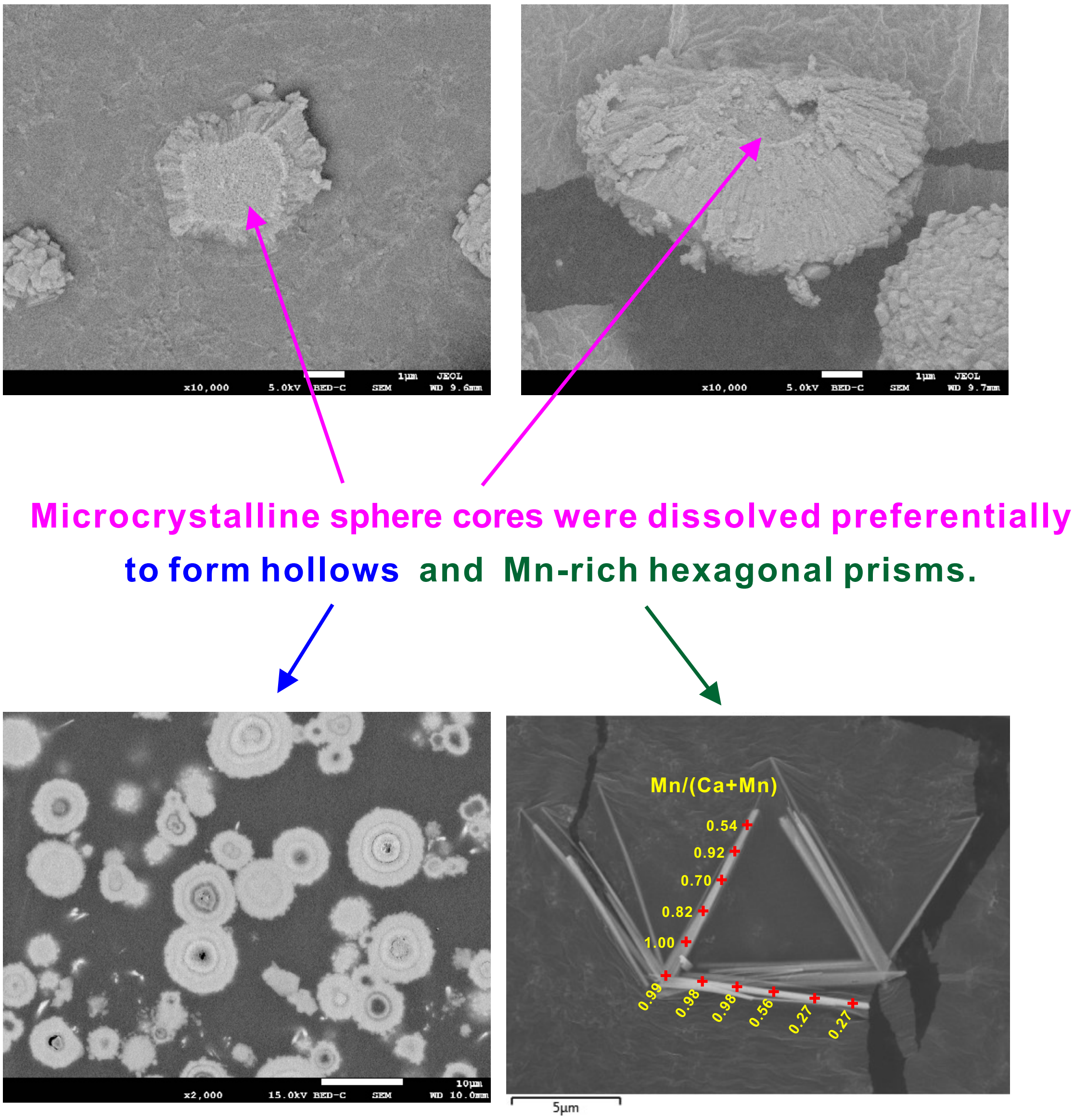

Appendix S5

BSE images of the equatorial sections and EDS analyses of the solid $(Ca_{0.68}Mn_{0.32})CO_3$ (CR-03) before and after dissolution in the air-saturated water for 300d to show that the microcrystalline sphere cores were dissolved preferentially to form hollows and Mn-rich hexagonal prisms.

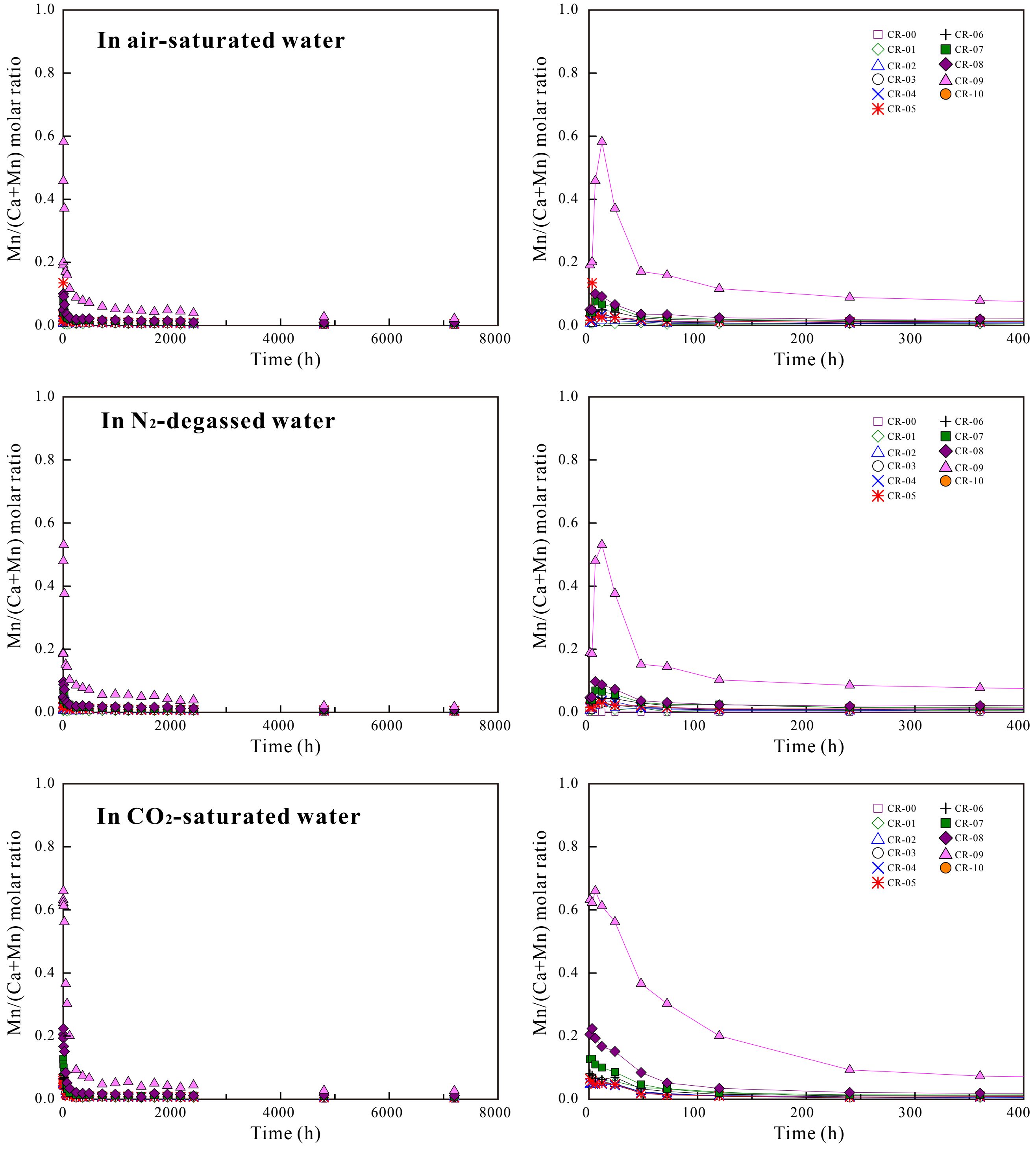

Appendix S6

Variation of the aqueous Mn/(Ca+Mn) mole ratios during the dissolution of the $(Ca_{1-x}Mn_x)CO_3$ solid solutions.

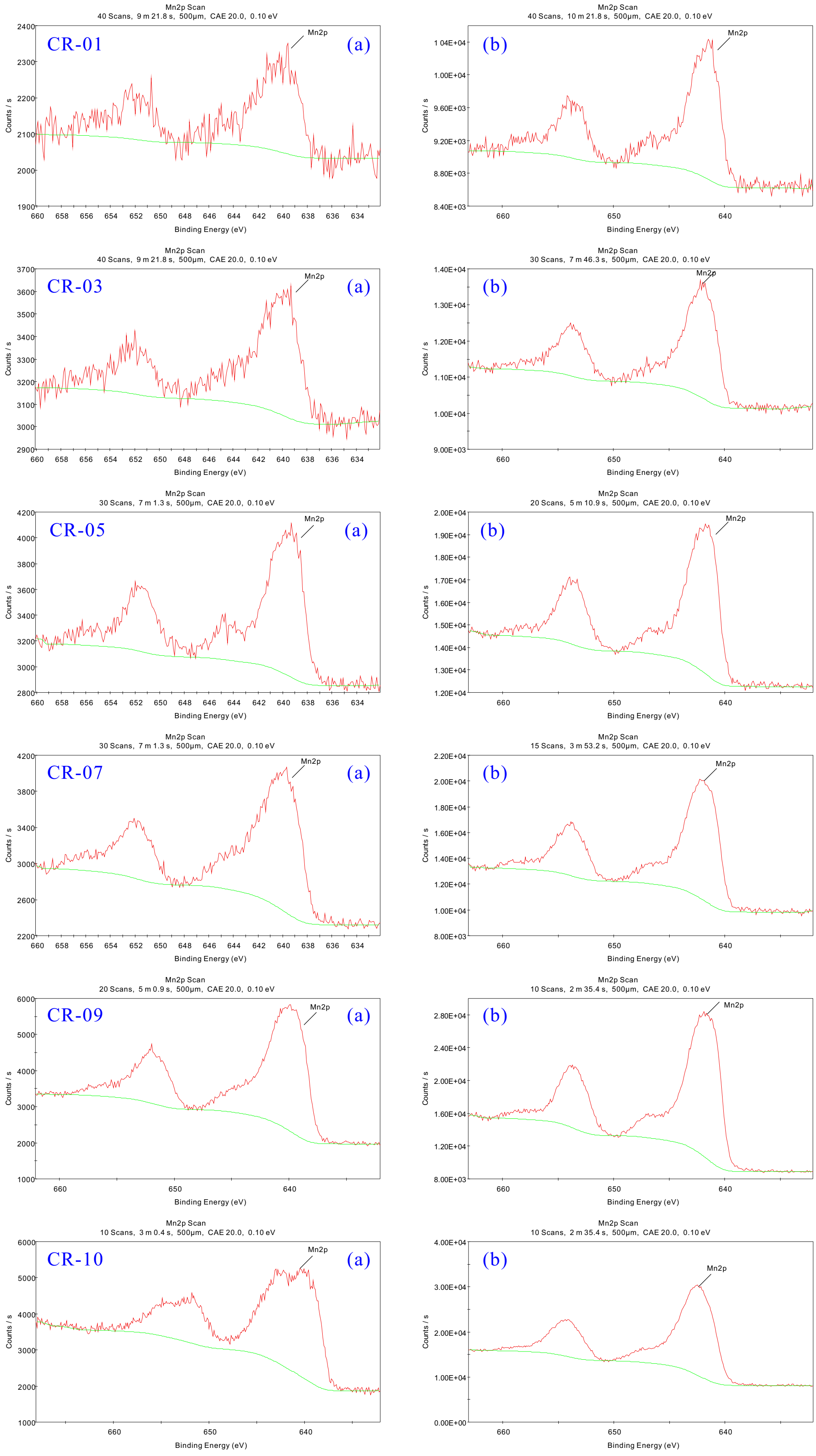

Appendix S7

XPS patterns of the $(Ca_{1-x}Mn_x)CO_3$ solid solutions
(a) before and (b) after dissolution in air-saturated water for 300 d.

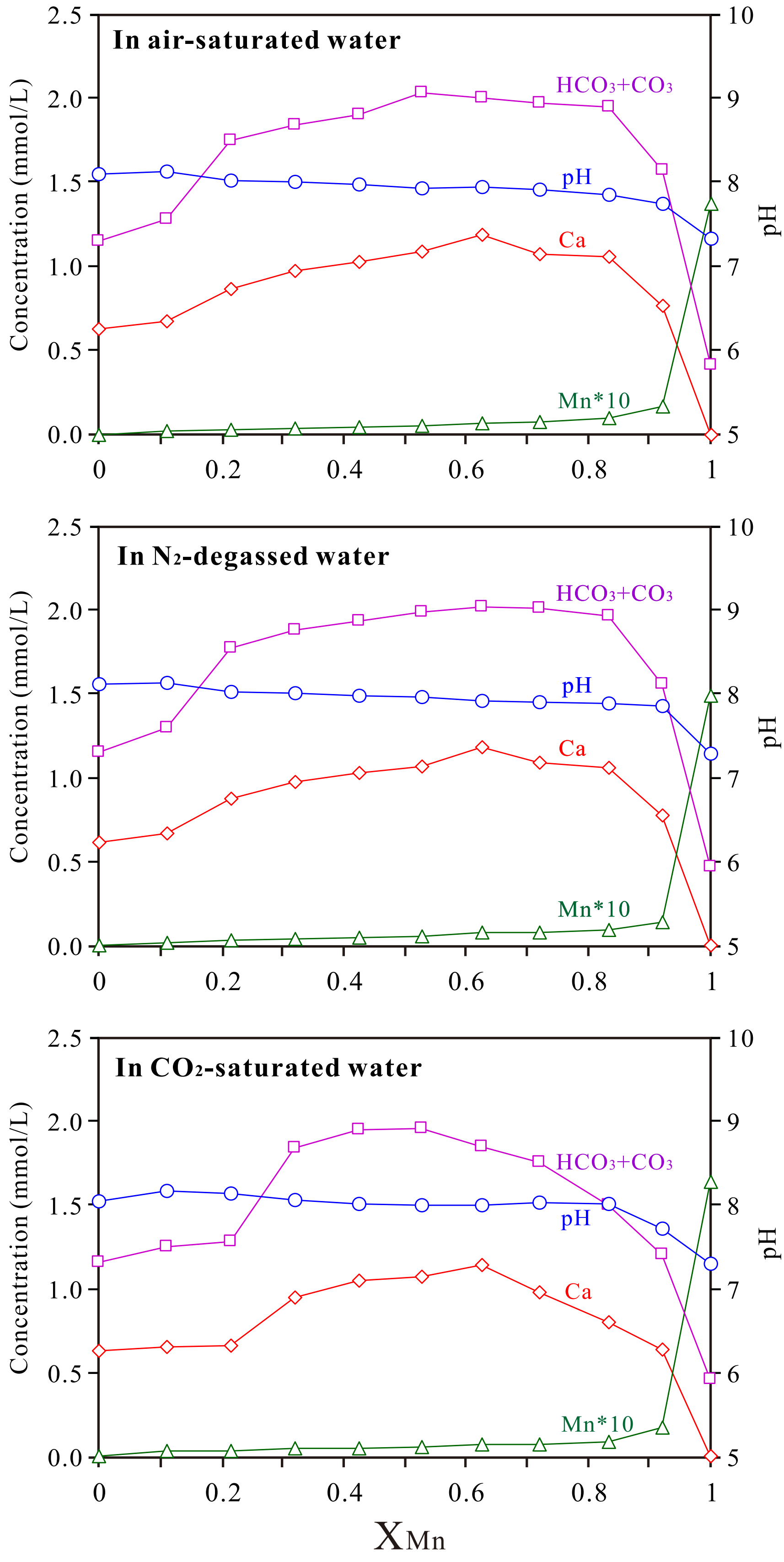

Appendix S8

Dependence of the aqueous components at the experimental end (7200h) on X_{Mn} of $(Ca_{1-x}Mn_x)CO_3$.

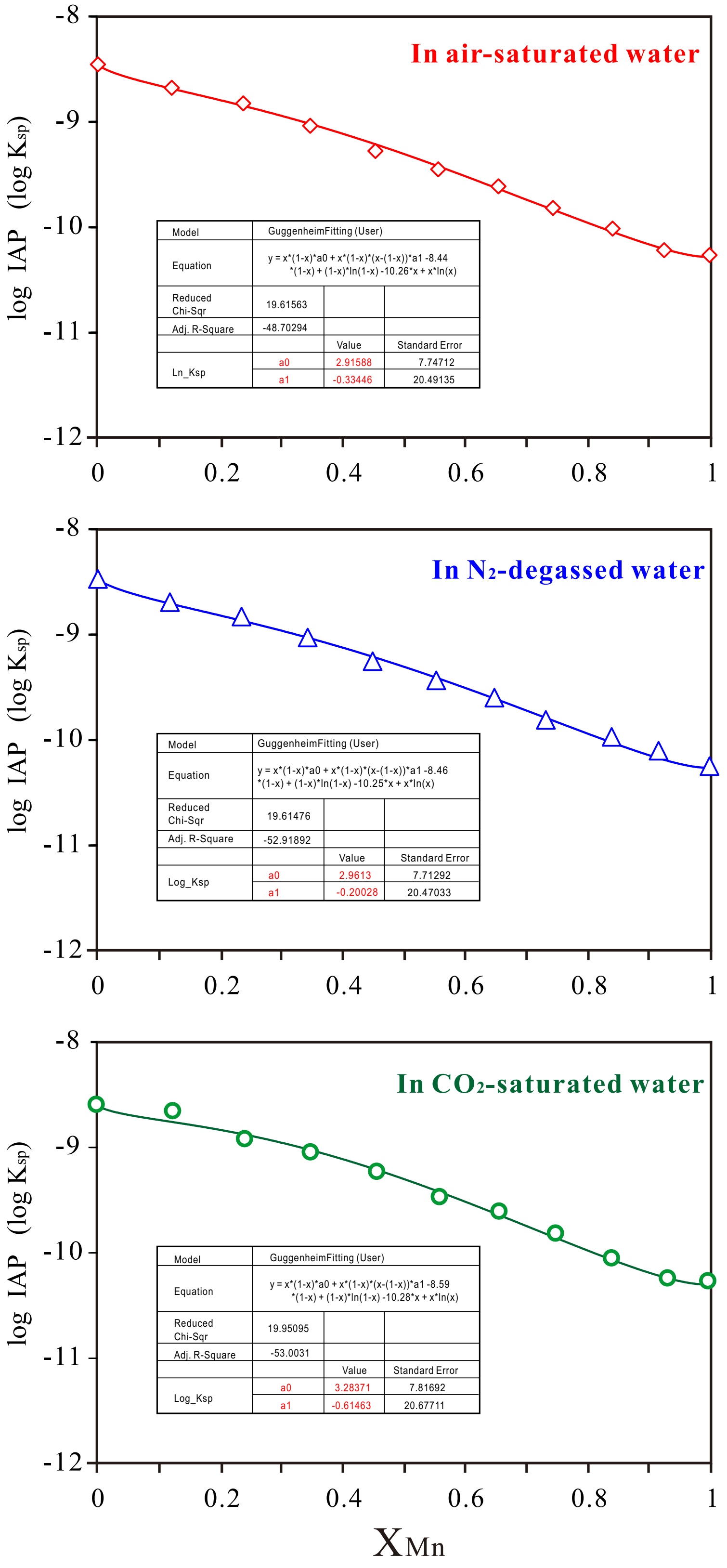

Appendix S9

Estimation of the Guggenheim coefficients for the non-ideal $(Ca_{1-x}Mn_x)CO_3$ solid solutions.

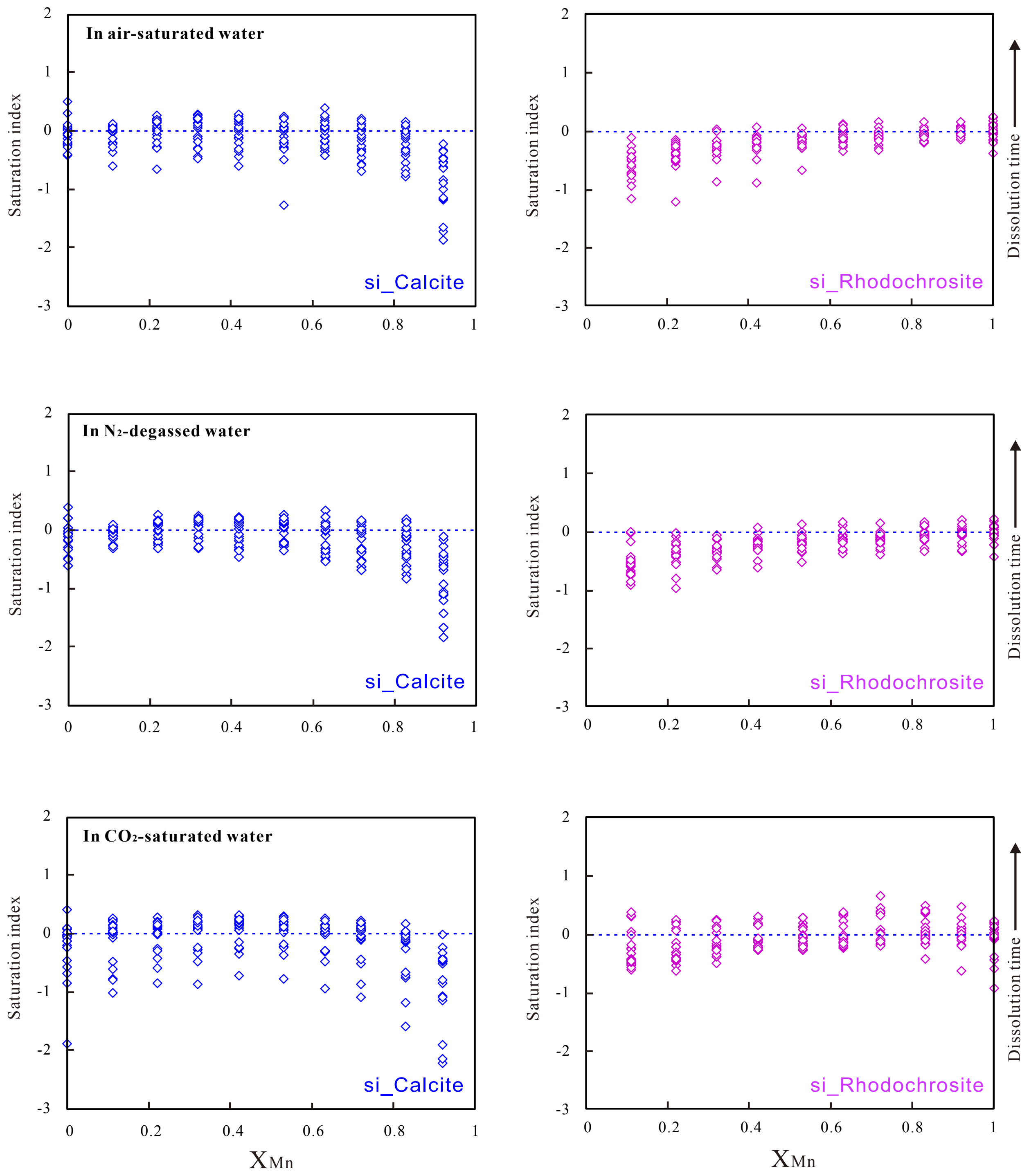

Appendix S10

Saturation indexes for calcite and rhodochrosite during the dissolution of the $(Ca_{1-x}Mn_x)CO_3$ solid solutions.
